# Proteolytic activity of the proteasome is required for female insect reproduction

**DOI:** 10.1098/rsob.200251

**Published:** 2021-02-24

**Authors:** Wei Wang, Rui-Rui Yang, Lu-Yao Peng, Lu Zhang, Yue-Lin Yao, Yan-Yuan Bao

**Affiliations:** ^1^ State Key Laboratory of Rice Biology and Ministry of Agriculture Key Laboratory of Molecular Biology of Crop Pathogens and Insect Pests, Institute of Insect Sciences, Zhejiang University, Hangzhou 310058, People's Republic of China; ^2^ School of Biological Science, University of Edinburgh, Edinburgh EH8 9AB, UK

**Keywords:** non-atpase regulatory subunit, proteolytic activity, reproduction, oocyte maturation, ovulation and hatching, *Nilaparvata lugens*

## Abstract

Non-ATPase regulatory subunits (Rpns) are components of the 26S proteasome involved in polyubiquitinated substrate recognition and deubiquitination in eukaryotes. Here, we identified 15 homologues sequences of *Rpn* and associated genes by searching the genome and transcriptome databases of the brown planthopper, *Nilaparvata lugens*, a hemipteran rice pest. Temporospatial analysis showed that *NlRpn* genes were significantly highly expressed in eggs and ovaries but were less-highly expressed in males. RNA interference-mediated depletion of *NlRpn* genes decreased the proteolytic activity of proteasome and impeded the transcription of *lipase* and *vitellogenin* genes in the fat bodies and ovaries in adult females, and reduced the triglyceride content in the ovaries. Decrease of the proteolytic activity of the proteasome via knockdown of *NlRpn*s also inhibited the transcription of *halloween* genes, including *NlCYP307A2*, *NlCYP306A2* and *NlCYP314A1*, in the 20-hydroxyecdysone (20E) biosynthetic pathway in the ovaries, reduced 20E production in adult females, and impaired ovarian development and oocyte maturation, resulting in reduced fecundity. These novel findings indicate that the proteolytic activity of the proteasome is required for female reproductive processes in *N. lugens*, thus furthering our understanding of the reproductive and developmental strategies in insects.

## Graphical abstract

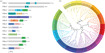

## 1. Introduction

The brown planthopper, *Nilaparvata lugens* Stål (Hemiptera: Delphacidae), is one of the most threatening pests of rice crops in tropical Asia and southern China [1,2]. *Nilaparvata lugens* possess high fecundity, enabling intense population outbreaks in optimal environmental conditions [3]. Offspring production and fitness are directly influenced by oogenesis and ovulation in oviparous insects [4]. The mechanisms regulating fecundity in insects have attracted interest for decades, and many genes, including *vitellogenin* (*Vg*) and *insulin* associated with nutritional metabolism, and *halloween* genes involved in ecdysteroid biosynthesis, are known to play important roles in the regulation of oocyte maturation and embryonic development in most insect species [1,5–12]. Progress has been achieved in understanding the molecular mechanisms governing female reproduction in *N. lugens* in recent years. The *DICER1* gene, which is mostly responsible for microRNA precursor processing, was demonstrated to be important for oocyte maturation in the telotrophic ovary [13], while the female-specific gene for *bicaudal-C* was essential for oogenesis [14]. The *Krüppel homologue 1* and *Broad-Complex* genes regulate ovarian development and jointly determine the number of ovarioles [15], while the *mucin*-*like* gene and eggshell-associated *NlChP38* genes are specifically expressed in the follicular cells and are vital for ovulation [16,17]. We previously demonstrated the need for the *pancreatic lipase-related protein 2* gene in oocyte maturation and development [18]. In addition to the characterized genes, other currently unknown genes may also be involved in fecundity. We previously sequenced the *N. lugens* genome and transcriptome in relation to developmental stage and tissue specificity [2,19–22]. The subsequent annotated database enabled us to identify potential fecundity-related genes in this insect species. In the present study, we conducted a genome- and transcriptome-wide search and identified a group of genes encoding non-ATPase regulatory subunits and associated proteins of the 26S proteasome, which were highly expressed in the ovaries of adult females and in eggs laid in rice leaf sheaths.

The 26S proteasome is a large ATP-dependent multiprotein complex and the major proteolytic machine in eukaryotic cells. It functions primarily to maintain protein homeostasis by eliminating damaged or misfolded proteins via the ubiquitin–proteasome system [23]. Eukaryotic proteostasis plays a key role in most cellular processes, including DNA damage repair, transcription, signal transduction, cell cycle progression, apoptosis, ageing and disease [24,25]. The 26S proteasome is composed of the 19S regulatory particle (RP) and the 20S proteolytic core particle (CP). The CP has structurally and mechanistically well-characterized proteolytic activities [26], while the RP is responsible for the recognition, deubiquitylation, unfolding and translocation of substrates into the CP for proteolysis [27]. The RP can be separated into two sub-structures: a base complex that can associate with the CP, and a peripheral lid complex [24,28]. In budding yeast, the base comprises six ATPase regulatory subunits and three non-ATPase subunits (Rpns1, 2 and 13), and an additional cofactor Rpn10 [24,29], of which Rpn1 and Rpn2 are the two largest non-ATPase subunits that function as a scaffold, presumably stabilizing the RP [28,30]. Rpn10 and Rpn13 were reported to act as ubiquitin receptors to bind polyubiquitinated proteins [28]. The lid is composed of eight different Rpn subunits (Rpns3, 5–9, 11 and 12) and a small acidic protein, Sem1 [27]. The major function of the lid is to serve as a specialized isopeptidase coupling substrate deubiquitination [31]. Rpn11 is a MPN+/JAMM (JAB1/MPN/Mov34 metalloenzyme)-domain-containing metalloprotease with deubiquitinase activity to remove ubiquitin chains from substrate proteins and to allow efficient protein degradation [27]. Rpn11 and its binding partner Rpn8 form a heterodimer in the lid, which is probably conducive to translocation-coupled substrate deubiquitination [32]. The Rpns3, 5–7, 9 and 12 subunits contain proteasome-COP9/signalosome-eIF3 (PCI) domains, and the Rpn15 subunit, a small acidic protein with neither PCI nor MPN domains, are thought to play scaffold roles in the lid [27,33,34]. Rpns have been extensively described in structural, biochemical and bioinformatics terms. The physiological functions for individual Rpns have been determined in yeast, *Drosophila* and *Caenorhabditis elegans* [31,35–38]. For instance, *C. elegans* RPN-12 is required for reproduction [35]. Yeast Rpn11 has metalloisopeptidase activity [31]. *Drosophila* Rpn11 is a suppressor of progressive neurodegeneration [38]. However, systematic assessment of the *in vivo* physiological functions for Rpns has not been well studied in higher eukaryotes such as most insect species. We are interested in studying the biological significance of Rpns in the reproduction mechanisms in a Hemipteran model insect *N. lugens* because its whole genome sequence has been elucidated [22] and is susceptible to RNA interference (RNAi) [12,39–45].

In this study, we identified 15 homologues genes of *Rpn* and associated chaperones in *N. lugens* by searching the genome and transcriptome databases. We investigated the functional roles of these genes in *N. lugens* using RNAi. Knockdown of the *NlRpn* genes notably reduced the proteolytic activity of the proteasome which then downregulated the transcript levels of several *lipase* and *Vg* genes in the fat bodies and the ovaries, decreased the triglyceride content of the ovaries, and resulted in remodelling of lipid droplets in the developing oocytes. In addition, decrease of the proteolytic activity via knockdown of *NlRpn*s downregulated the transcript levels of *halloween* genes in the 20-hydroxyecdysone (20E) biosynthetic pathway in the ovary and reduced 20E synthesis throughout the body in adult females, leading to reproductive failure. Studies have established the 26S proteasome as an important regulator of transcription through proteolytic and non-proteolytic activities [46,47]. Genetic or chemical inhibition of proteasome function is known to result in significant changes in gene expression patterns [46–49]. Genes involved in mitochondrial function, stress response and protein degradation were upregulated, while ribosomal protein genes, mating genes and amino acid metabolism genes were downregulated in yeast [46]. In this study, our experiments revealed depletion of *Rpn*s by RNAi significantly decreased the proteolytic activity of proteasome, which then affects the gene transcription during reproduction processes in *N. lugens*.

## 2. Results

### 2.1. Bioinformatics analysis of *Rpn* and associated genes in *Nilaparvata lugens*

**Figure 1.**
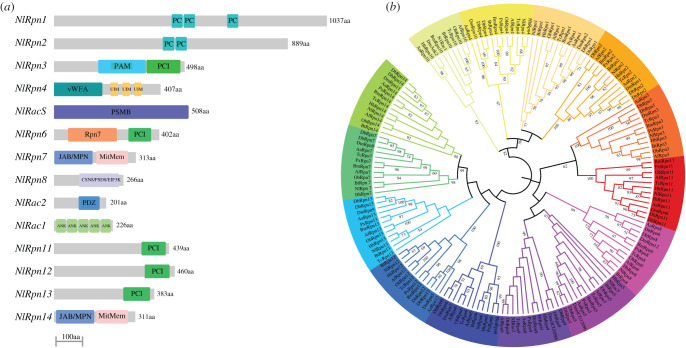
Bioinformatics analysis of *Rpn* and *assembly chaperone* genes. (*a*) Domain structures of NlRpn and NlRac proteins. Conserved domains in the deduced amino acid sequences of NlRpns and NlRacs were determined using SMART (http://smart.embl.de/), Pfam (http://pfam.xfam.org/) and NCBI (http://www.ncbi.nlm.nih.gov/). Grey bars represent the number of amino acid residues and coloured boxes represent the characteristic domains. PC, proteasome/cyclosome repeat; PAM, PCI/PINT-associated module; PCI, proteasome-COP9/signalosome-eIF3; vWFA, von Willebrand factor type A; UIM, ubiquitin-interacting motif; PSMB, proteasome subunit beta type; Rpn7, 26S proteasome subunit Rpn7, known as the Rpn6 in higher eukaryotes; JAB/MPN, Jun kinase activation domain-binding protein and proteasomal subunits/Mpr1p-Pad1p-N-terminal; MitMem, C-terminal to the Mov24 region of the yeast proteasomal subunit Rpn11; CSN8/PSD8/EIF3K, COP9 signalosome/proteasome 26S non-ATPase subunit 8/eukaryotic translation initiation factor 3 subunit; PDZ, PSD95-DLG-ZO1/2; ANK, ankyrin repeat. The GenBank accession numbers for the *NlRpn* and *NlRac* sequences were as follows: *NlRpn1* (MT755974), *NlRpn2* (MT755975), *NlRpn3* (MT755976), *NlRpn4* (MT755977), *NlRac5* (MT755978), *NlRpn6* (MT755979), *NlRpn7* (MT755980), *NlRpn8* (MT755981), *NlRac2* (MT755982), *NlRac1* (MT755983), *NlRpn11* (MT755984), *NlRpn12* (MT755985), *NlRpn13* (MT755986) *and NlRpn14* (MT755987). (*b)* Phylogenetic analysis of insect RPNs and assembly chaperones. The phylogenetic tree was constructed based on the deduced amino acid sequences of 165 RPNs and assembly chaperones from 12 insect species by the maximum-likelihood method using Mega X (http://www.megasoftware.net/). Phylogenetic relationships were determined using the Jones–Taylor–Thornton for amino acid substitution model. Bootstrap analysis was set for values of 1000 trials and bootstrap values greater than 50% are shown on each node of the tree. *Nl*, *N. lugens*; *Hh*, *Halyomorpha halys*; *Bt*, *Bemisia tabaci*; *Px*, *Plutella xylostella*; *Bm*, *Bombyx mori*; *Ob*, *Ooceraea biroi*; *Af*, *Apis florea*; *Tc*, *Tribolium castaneum*; *Aa*, *Aedes aegypti*; *Db*, *Drosophil busckii*; *Dh*, *Drosophila hydei*; *Dm*, *Drosophila melanogaster*.

We identified 15 genes encoding Rpns and associated proteins by searching the *N. lugens* genomic and transcriptomic databases (electronic supplementary material, table S1). The deduced *N. lugens* amino acid sequences had high identities with their homologues in human, yeast and most insect species. The predicted domains were well conserved. Here, we follow the nomenclature strategy of human *Rpn* subunits to name the *N. lugens* homologues as *NlRpn1*-*4*, *NlRpn6*-*8*, *NlRpn11*-*14* and *NlADRM1* (table 1). To distinguish *Rpn* subunits and assembly chaperones, we name three *N. lugens* assembly chaperones as *NlRac1*, *NlRac2* and *NlRac5*. The two largest proteins, NlRpn1 and NlRpn2, were composed of 1037 and 889 amino acid residues containing three and two characteristic proteasome/cyclosome repeat (PC) domains, respectively (figure 1*a*). NlRpn3, NlRpn6, NlRpn11, NlRpn12 and NlRpn13 consisted of 383–498 amino acids and shared a conserved PCI domain. NlRpn3 and NlRpn6 also contained a PCI/PINT-associated module (PAM) and an Rpn7 domain (26S proteasome subunit Rpn7, known as the Rpn6 in higher eukaryotes) (pfam10602), respectively. NlRpn4 contained a von Willebrand factor type A (vWFA) and three ubiquitin-interacting motif (UIM) domains. NlRpn8 had only a COP9 signalosome/proteasome 26S non-ATPase subunit 8/eukaryotic translation initiation factor 3 subunit (CSN8/PSD8/EIF3K) domain. NlRpn7 and NlRpn14 shared similar amino acid compositions and domain organizations comprising a Jun kinase activation domain-binding protein and proteasomal subunits/Mpr1p-Pad1p N-termini (JAB/MPN) domain at the N-terminus and a Mov24 region of proteasomal subunit Rpn11 (MitMem region) at the C-terminus. NlRac1 was predicted to be a small protein, containing 226 amino acid residues and composed of five ankyrin repeat domains. NlRac2 was also a small protein, with 201 amino acid residues containing a PSD95-DLG-ZO1/2 (PDZ) domain. NlRac5 had a proteasome subunit beta type domain (PSMB: pfam10508).

**Table 1.** The nomenclature of the *RPN* genes in the different organisms. — indicates no orthologous genes.

*N. lugens*	*H. sapiens*	*many insects*	*S. cerevisiae*	*D. melanogaster*	Function
*NlRpn1*	*PSMD1*	*Rpn1*	*ScRpn2*	*DmRpn2*	
*NlRpn2*	*PSMD2*	*Rpn2*	*ScRpn1*	*DmRpn1*	
*NlRpn3*	*PSMD3*	*Rpn3*	*ScRpn3*	*DmRpn3*	
*NlRpn4*	*PSMD4*	*Rpn4*	*ScRpn10*	*DmRpn10*	
*NlRac5*	*PSMD5*	*Rpn5*	*—*	*Dmel_CG12096*	assembly chaperone
*NlRpn6*	*PSMD6*	*Rpn6*	*ScRpn7*	*DmRpn7*	
*NlRpn7*	*PSMD7*	*Rpn7*	*ScRpn8*	*DmRpn8*	
*NlRpn8*	*PSMD8*	*Rpn8*	*ScRpn12*	*DmRpn12*	
*NlRac2*	*PSMD9*	*Rpn9*	*Nas2p*	*Dmel_CG9588*	assembly chaperone
*NlRac1*	*PSMD10*	*Rpn10*	*Nas6p*	*Ank2*	assembly chaperone
*NlRpn11*	*PSMD11*	*Rpn11*	*ScRpn6*	*DmRpn6*	
*NlRpn12*	*PSMD12*	*Rpn12*	*ScRpn5*	*DmRpn5*	
*NlRpn13*	*PSMD13*	*Rpn13*	*ScRpn9*	*DmRpn9*	
*NlRpn14*	*PSMD14*	*Rpn14*	*ScRpn11*	*DmRpn11*	
*NlADRM1*	*ADRM1*	*—*	*ScRpn13*	*DmRpn13*	
*—*	*SEM1*	*—*	*Sem1*/*HOD1*	*—*	

We constructed a phylogenetic tree based on the complete amino acid sequences available in the GenBank database in order to understand the evolutionary relationships among NlRpns and associated assembly chaperones and their homologues in other insect species. Phylogenetic analysis indicated that the insect Rpns and assembly chaperones were separated into individual branches (figure 1*b*). Rpn1 and Rpn2 had the characteristic PC domains, were clustered together and were closely related to Rpn4 and Rpn10/Rac1, and formed an independent cluster divergent from all other Rpns. The Rpn5/Rac5, Rpn9/Rac2 and Rpn8 branches were closely related, clustered together, and diverged from Rpn3 and Rpn11 branches with a conserved PCI domain. The Rpn7 and Rpn14 branches that shared JAB/MPN and MitMem domains were closely related. The Rpn6, Rpn12, and Rpn13 branches had a PCI domain and were clustered together. Most branches of *N. lugens* RPNs and assembly chaperones showed the closest phylogenetic relationships with homologues in Hemiptera such as *Halyomorpha halys* and *Bemisia tabaci*, but were distant from their homologues in Diptera, such as *Aedes aegypti* and *Drosophila hydei*. The nomenclature of the *Rpn* and *assembly chaperone* genes varies among organisms. The names of the *Rpn* genes in most insect species, except *Drosophila melanogaster*, are consistent with the standardized names of the human *Rpn*s, which differ from the gene names in the yeast *Saccharomyces cerevisiae* and *D*. *melanogaster* (table 1). *ScRpn13* in *S. cerevisiae* and *DmRpn13* in *D. melanogaster* are homologous to ADRM1 in humans, which is a 19S RP subunit that does not follow the human PSMD naming strategy. In *N. lugens*, we name the homologue of *ADRM1*/*ScRpn13*/*DmRpn13* as *NlADRM1*. *NlRac1* and *NlRac2*, the RP assembly chaperones, are shown to be the homologues of *PSMD10*/*ScNas6p*/*DmAnk2*/*Rpn10* and *PSMD9*/*ScNas2p*/*Deml_CG9588/Rpn9* in humans, yeast, *Drosophila* and most insect species. *NlRac5* is the homologue of *PSMD5*/*Deml_CG12096*/*Rpn5* in humans, *Drosophila* and most insect species, but is absent in yeast. Intriguingly, homologues of human and yeast *Sem1*/*HOD1* are lacking in most insects.

### 2.2. Temporospatial expression patterns of *NlRpn* and *NlRac* genes

**Figure 2.**
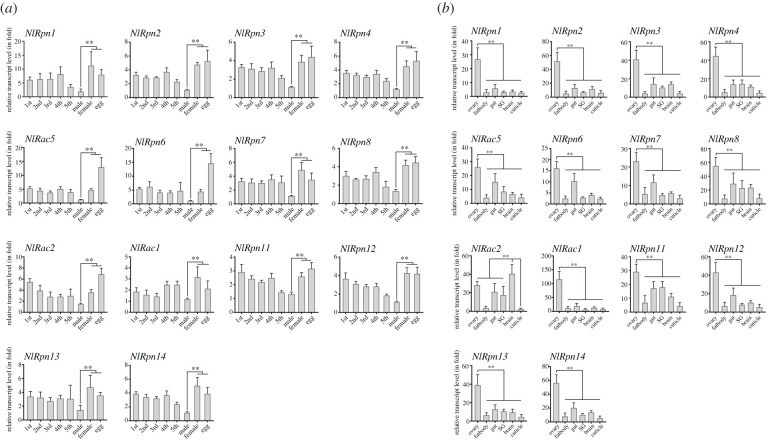
Temporospatial expression patterns. (*a*) Developmental stage-specific expression analysis (in fold) of *NlRpns* and *NlRacs*. Total RNAs were extracted from laid eggs, nymphs and adults (*n* = 100 laid eggs, 20–80 nymphs and 20 adults). 1st, 2nd, 3rd, 4th and 5th refer to 1st–5th instar nymphs. One microgram of starting RNA was used for reverse transcription in a 20 µl reaction. Two microlitres of the first-strand cDNA (diluted 10 times) was analysed in each 20 µl reaction by qRT-PCR. The relative transcript levels of each gene in each developmental stage were normalized using the *N. lugens 18S rRNA* or *β-actin* threshold cycle (*C_t_*) values that were obtained from reactions run on the same plate. In each assay, the transcript level was normalized to the lowest transcript level, which was arbitrarily set at one. (*b*) Tissue-specific expression analysis (in fold) of *NlRpns* and *NlRacs*. Total RNAs were extracted from the ovary, fat body, gut, salivary gland (SG), brain and cuticle of adult female *N. lugens* (*n* = 50–100). Relative transcript levels of the target genes in each tissue were determined by qRT-PCR as described above. Statistical analysis was performed using GraphPad Prism 8 software (San Diego, CA, USA). Three independent biological replicates (mean ± standard deviation) were conducted and relative transcript levels in each sample were measured using the ΔΔ*C_t_* method. **p < 0.01 between indicated developmental stages or tissues (Student's *t*-test).

We investigated the functional roles of the *NlRpn* and *NlRac* genes by examining their expression patterns throughout development and in different tissues using quantitative real-time polymerase chain reaction (qRT-PCR). These genes displayed similar developmental stage specificities, with the highest transcript levels in eggs and/or adult females and the lowest levels in adult males (figure 2*a*). *NlRpn1*, *NlRpn7*, *NlRac1*, *NlRpn13* and *NlRpn14* transcripts were detected at higher levels in adult females than in eggs, while *NlRac5*, *NlRpn6* and *NlRac2* transcripts were detected at much higher levels in eggs than in adult females. The other *Rpn* transcripts, including *NlRpn2*, *NlRpn3*, *NlRpn4*, *NlRpn8*, *NlRpn11* and *NlRpn12* were expressed at similarly high levels in both eggs and adult females. These observations suggest that *NlRpn* and *NlRac* genes may have physiological functions in female reproduction and egg development. We subsequently investigated their tissue specificity in adult females. These genes, except *NlRac2*, displayed the highest transcript levels in ovaries and the lowest levels in fat bodies and cuticles among all the tested tissues (figure 2*b*). Notably, *NlRac2* showed the highest transcript levels in the brain followed by ovaries. These results further suggest that *NlRpn* and *NlRac* genes play vital roles in ovarian tissues in adult females, and may have additional functions in *N. lugens*.

### 2.3. Investigation of physiological functions of *NlRpn* and *NlRac* genes by RNA interference

**Figure 3.**
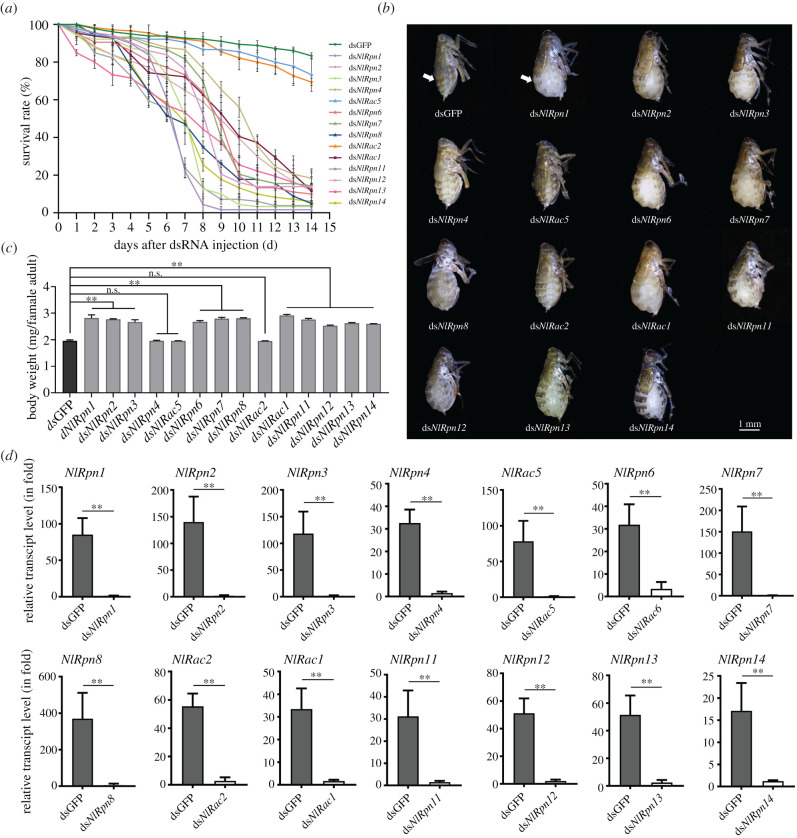
Phenotypic observation of RNAi targeting *NlRpns* and *NlRacs*. (*a*) Dynamic analysis of survival rates of *N. lugens*. Newly emerged females were injected with each ds*NlRpn* or ds*NlRac*, respectively, and observed for phenotypic variations at 24 h intervals for 14 days. dsGFP was injected as a negative control to determine the non-specific effects of dsRNA. Three independent biological replicates were conducted for each treatment (mean ± standard deviation; *n* = 60 females). (*b*) Morphology was observed on the 7th day after RNAi with each ds*NlRpn* or ds*NlRac* in the newly emerged females. Arrowheads indicate intersegmental membranes. Scale bar, 1.0 mm. (*c*) Body weights of females were measured on the 7th day after RNAi with each ds*NlRpn* or ds*NlRac* in the newly emerged females. Three independent biological replicates were conducted for each treatment (mean ± standard deviation; *n* = 20 females). **p < 0.01 between dsGFP and ds*NlRpn* or ds*NlRac* treatments (Student's *t*-test); ns: no significant difference. (*d*) Determination of RNAi efficiency. Total RNAs were extracted from *N. lugens* on the 3rd day after RNAi and transcript levels of each *NlRpn* and *NlRac* were analysed by qRT-PCR, as described in figure 2. One microgram of each RNA sample was used for reverse transcription in a 20 µl reaction. Two microlitres of the first-strand cDNA (diluted 10 times) was analysed in each 20 µl reaction by qRT-PCR. The relative transcript levels of each *NlRpn* and *NlRac* gene in ds*NlRpn*, ds*NlRac* and dsGFP treatments were normalized using the *N. lugens 18S rRNA* or *β-actin* *C_t_* values. In each assay, the transcript level was normalized to the lowest transcript level, which was arbitrarily set at one. Results of triplicate experiments are shown with standard deviations. **p < 0.01 compared with dsGFP-injected controls (Student's *t*-test).

We investigated the physiological functions of *NlRpn* and *NlRac* genes by silencing their expression in *N. lugens in vivo* using an RNAi approach. Newly emerged females were individually microinjected with each target double-stranded RNA (dsRNA) and phenotypic variations were observed at 24 h intervals following RNAi. Knockdown of *NlRpn* and *NlRac1* transcription generated apparently lethal phenotypes. Following RNAi, greater than 70% of individuals in ds*NlRpn1*-, ds*NlRpn2*-, ds*NlRpn3*-, ds*NlRpn4*-, ds*NlRpn6*-, ds*NlRpn7*-, ds*NlRpn8*-, ds*NlRac1*-, ds*NlRpn11*-, ds*NlRpn12*-, ds*NlRpn13*- or ds*NlRpn14*-injected group survived at 3 days post-injection (dpi), but these rates decreased at 4 dpi and declined dramatically to less than 40% at 11 dpi and to less than 20% at 14 dpi (figure 3*a*). By contrast, knockdown of *NlRac5* and *NlRac2* had no significant effect on the survival of *N. lugens* throughout the test period, similar to controls treated with double strand green fluorescent protein (dsGFP). Interestingly, adult females treated with ds*NlRpn*s and ds*NlRac*s, except *NlRpn4*, *NlRac5* and *NlRac2*, showed an abnormal phenotype from 7 dpi, with an obviously inflated abdomen and stretched intersegmental membranes in the tergum (figure 3*b*). Knockdown of *NlRpn*s and *NlRac*s, except *NlRpn4*, *NlRac5* and *NlRac2*, also significantly increased female body weight by approximately 29–48% compared with dsGFP-injected controls at 7 dpi (figure 3*c*). qRT-PCR analysis confirmed that transcript levels of each *NlRpn* or *NlRac* gene were notably reduced in RNAi-injected insects compared with those in dsGFP-injected controls at 3 dpi (figure 3*d*).

### 2.4. Investigation of the effects of specific *NlRpn* or *NlRac* knockdown on other *NlRpn* and *NlRac* gene expression

**Figure 4.**
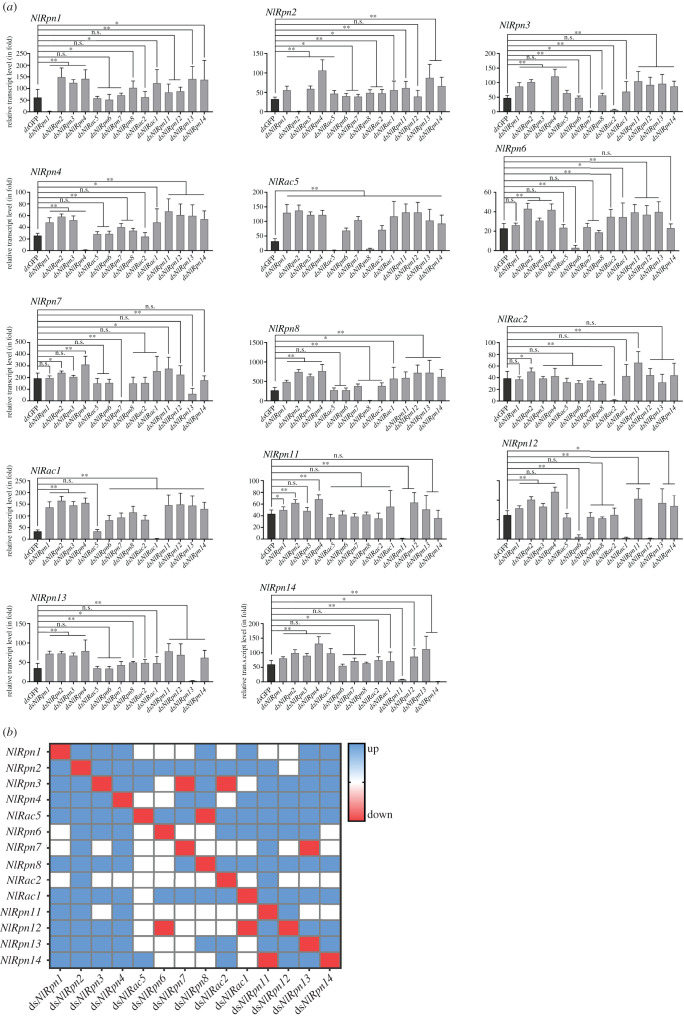
RNAi effects depleting specific *NlRpn* and *NlRac* genes on other *NlRpn* and *NlRac* gene expression. (*a*) Determination of transcript level variations of *NlRpn* and *NlRac* genes. RNAi was performed by microinjecting each specific ds*NlRpn* or ds*NlRac* into the newly emerged female adults. Total RNAs were extracted from the whole bodies of the insects on the 5th day after RNAi (*n* = 10 adults). One microgram of starting RNA was used for reverse transcription in a 20 µl reaction. Two microlitres of the first-strand cDNA (diluted 10 times) was analysed in each 20 µl reaction by qRT-PCR. The relative transcript levels of each gene in each RNAi treatment with specific dsRNA were normalized using the *N. lugens 18S rRNA* or *β-actin* *C_t_* values as described in figure 2. In each assay, the transcript level was normalized to the lowest transcript level, which was arbitrarily set at one. Statistical analysis was performed using GraphPad Prism 8 software (San Diego, CA, USA). Three independent biological replicates (mean ± standard deviation) were conducted and relative transcript levels were measured using the ΔΔ*C_t_* method. **p < 0.01, *p < 0.05 between dsGFP and a specific dsRNA treatments (Student's *t*-test). *NlRpn* and *NlRac* in the upper-left of each graph refer to the gene expression; ds*NlRpn* and ds*NlRac* of *x*-axis refer to specific dsRNA treatment. (*b*) Heatmap of transcript level variations of *NlRpn* and *NlRac* genes. Heatmap was plotted using GraphPad Prism 8 software based on the above qRT-PCR data from three biological replicates. *NlRpn* and *NlRac* of *y*-axis refer to the genes; ds*NlRpn* and ds*NlRac* of *x*-axis refer to specific dsRNA treatment. Red and blue refer to significantly downregulated and upregulated transcript levels of the genes after depleting specific *NlRpn* or *NlRac* gene expression. White refers to no significant change at the transcript levels.

To understand the effects of specific *NlRpn* or *NlRac* knockdown on other *NlRpn* and *NlRac* gene expression, we examined the transcript level variations of *NlRpn* and *NlRac* genes in the adult females on the 5th day after emergence upon knockdown of a specific *NlRpn* or *NlRac* gene expression. As a result, knockdown of individual *NlRpn1*, *NlRpn2*, *NlRpn3*, *NlRpn4*, *NlRac5*, *NlRpn12* or *NlRpn14* genes significantly upregulated or did not change the transcript levels of other *NlRpn* and *NlRac* genes (figure 4*a,b*). Knockdown of individual *NlRpn6*, *NlRpn7*, *NlRpn8*, *NlRac2*, *NlRac1*, *NlRpn11* or *NlRpn13* genes also significantly upregulated or did not change the transcript levels of *NlRpn* and *NlRac* genes, but decreased the transcript levels of several genes. Knockdown of *NlRpn6* or *NlRac1* significantly reduced *NlRpn12* transcript levels; knockdown of *NlRpn7* or *NlRac2* significantly reduced *NlRpn3* transcript levels; while knockdown of *NlRpn8*, *NlRpn11* or *NlRpn13* significantly reduced *NlRac5*, *NlRpn14* or *NlRpn7* transcript levels, individually.

### 2.5. Effect of silencing *NlRpn* and *NlRac* on ovarian morphology in *Nilaparvata lugens* adult female

**Figure 5.**
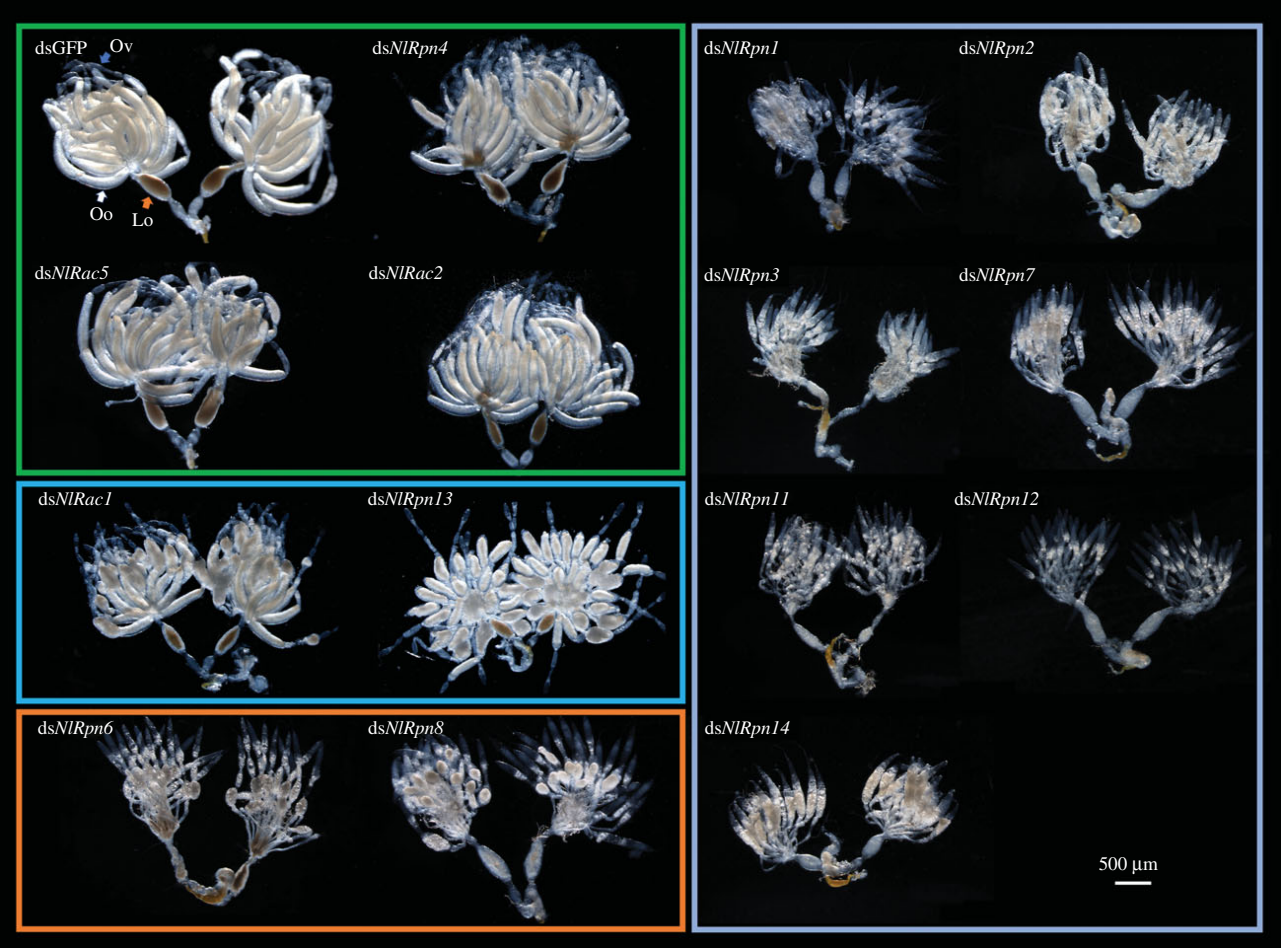
Effect of *NlRpn* and *NlRac* RNAi on ovarian development and oocyte maturation. Ovaries were dissected from adult females on the 5th day after RNAi. Insects injected with dsGFP were used as negative controls for the non-specific effects of dsRNA. Oo, oocyte; Ov, ovariole; Lo, lateral oviduct. Scale bar, 500 µm.

Because *NlRpn* and *NlRac* genes were highly expressed in *N. lugens* ovaries, we investigated the ovarian phenotypes resulting from the knockdown of each *NlRpn* or *NlRac* in newly emerged adult females. The ovaries were dissected and observed on the 5th day after emergence. Adult females injected with ds*NlRpn4*, ds*NlRac5* or ds*NlRac2* showed normal ovaries with regular, banana-shaped mature oocytes, as in dsGFP-injected females (figure 5). By contrast, females injected with other ds*NlRpn*s and ds*Rac1* displayed apparently abnormal ovaries, with no or malformed oocytes with irregular shapes. Ds*NlRac1*- and ds*NlRpn13*-injected females had chubby, globose oocytes in the ovarioles, while ds*NlRpn6*- and ds*NlRpn8*-injected females had segmented ovarioles with malformed, round-shaped oocytes. Females injected with ds*NlRpn1*, ds*NlRpn2*, ds*NlRpn3*, ds*NlRpn7*, ds*NlRpn11*, ds*NlRpn12* or ds*NlRpn14* had no obviously segmented ovarioles in the ovaries, but had abnormal-looking immature oocytes containing large lipid droplets, loosely distributed in the ovarioles. The lateral oviducts of the ovaries also appeared milky white, compared with brown in dsGFP-injected females.

### 2.6. Investigation of lipid accumulation in ovaries

**Figure 6.**
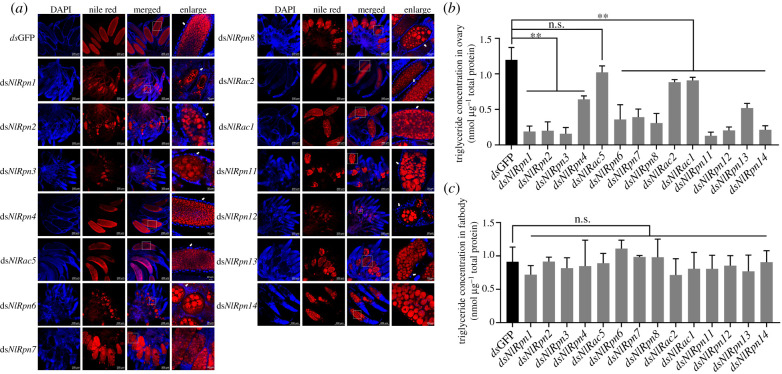
Investigation of lipid accumulation in ovaries. (*a*) Observation of lipid droplets in the ovaries after RNAi. Newly emerged females were injected with ds*NlRpn* or ds*NlRac* and the ovaries were isolated on the 5th day after emergence. Intracellular lipid droplets were stained by Nile red and nuclei in the follicular epithelial cells were stained by 4′,6-diamidino-2-phenylindole (DAPI), shown as red and blue fluorescence, respectively. dsGFP was used as a negative control. Enlarged images are shown in the right panels. White arrows indicate the nuclei of the follicular cells. Scale bars, 200 µm; scale bars of enlarged images, 20 µm. Measurement of triglycerides in the ovary and fat body of *N. lugens* females. Triglyceride contents of ovaries (*b*) and fat bodies (*c*) dissected from ds*NlRpn*- or ds*NlRac*-injected females on the 5th day after emergence. dsGFP-injected samples were used as controls. Triglyceride content (nmol triglyceride μg^−1^ total protein) was determined from three independent biological replicates (mean ± standard deviation; *n* = 5). **p < 0.01 between ds*NlRpn* or ds*NlRac* and dsGFP treatments (Student's *t*-tests); ns: no significant difference.

We monitored the morphological changes and distribution of lipid droplets in the ovaries of ds*NlRpn*- and ds*NlRac*-injected females using Nile red staining, which revealed abnormal sizes and uneven distribution of lipid droplets in oocytes after knockdown of *NlRpn* or *NlRac* transcript levels. dsGFP-injected ovaries included large numbers of small, round, evenly distributed lipid droplets in the oocytes (figure 6*a*). Uniformly distributed round lipid droplets with similar sizes were observed in the oocytes of females injected with ds*NlRpn4*, ds*NlRac5* and ds*NlRac2*, while the lipid droplets in the immature oocytes were remodelled and obviously larger in the ovaries of females injected with ds*NlRpn1*, ds*NlRpn2*, ds*NlRpn3*, ds*NlRpn6*, ds*NlRpn7*, ds*NlRpn8*, ds*NlRac1*, ds*NlRpn11*, ds*NlRpn12*, ds*NlRpn13* and ds*NlRpn14*. These large lipid droplets were surrounded by some medium-sized or small droplets. Interestingly, some immature oocytes in ds*NlRpn1*- and ds*NlRpn2*-injected females had lipid droplets distributed at the periphery of the oocytes. These observations implied that knockdown of most *NlRpn*s and *NlRac1* led to the remodelling of lipid droplets in oocytes. In addition, as shown in figure 6*a*, each oocyte is surrounded by a single layer of follicular epithelial cells with two nuclei in ovarioles in dsGFP-injected females, but the oocyte is surrounded by the disordered follicular cells that did not possess the typical two nuclei after knockdown of *NlRpn*s and *NlRac*s, except for *NlRpn4*, *NlRac5* and *NlRac2*, suggesting that most *NlRpn*s and *NlRac1* were involved in the accumulation of lipid droplets in oocytes and the development of follicular cells.

Based on the observation of abnormal lipid droplets in oocytes in ds*NlRpn*- and ds*NlRac*-injected females, we measured the triglyceride content, as a major component of lipid droplets, in female ovaries and fat bodies. The triglyceride contents in dsGFP-injected ovaries on the 5th day after emergence were 1.2 nmol µg^−1^ total protein (figure 6*b*). RNAi of *NlRpn*s and *NlRac*s, except *NlRac5*, significantly reduced the triglyceride contents of the ovaries by 24–57% in the case of ds*NlRpn4*, ds*NlRac2*, ds*NlRac1*, and ds*NlRpn13*-injected females, 67–74% in ds*NlRpn6*, ds*NlRpn7* and ds*NlRpn8*-injected females, and 82–89% in ds*NlRpn1*, ds*NlRpn2*, ds*NlRpn3*, ds*NlRpn11*, ds*NlRpn12* and ds*NlRpn14*-injected females, compared with dsGFP-injected controls. The triglyceride content in the fat bodies in dsGFP-injected females was 0.9 nmol µg^−1^ total protein (figure 6*c*), and was not significantly affected by RNAi with any *NlRpn* or *NlRac*. These results suggest that knockdown of *NlRpn* and *NlRac* affected the accumulation of triglycerides in oocytes in the ovaries, but not in fat body cells.

### 2.7. Changes in *lipase* gene expression in ovaries and fat bodies

**Figure 7.**
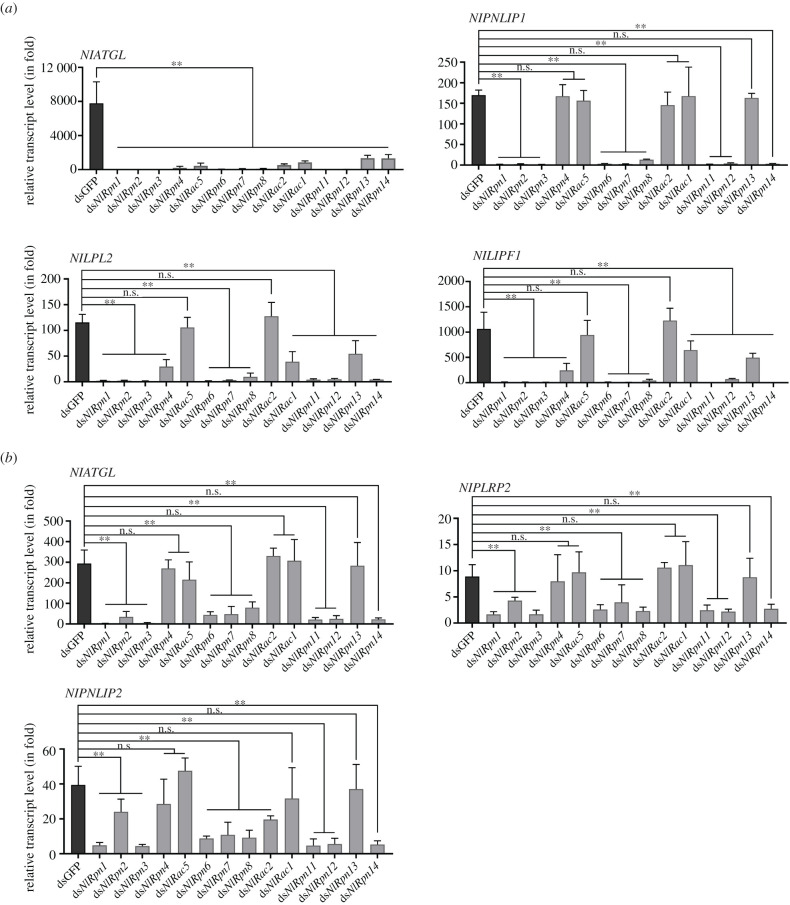
Analysis of *lipase* transcript levels (in fold) after knockdown of *NlRpn*s and *NlRac*s. Transcript levels of *lipase* genes in ovaries (*a*) and fat bodies (*b*) from ds*NlRpn*- or ds*NlRac*-injected females on the 5th day after emergence. dsGFP-injected samples were used as controls. Relative transcript levels of *lipase* genes in each treatment were determined by qRT-PCR. Three independent biological replicates (mean ± standard deviation; *n* = 10) were conducted as described in figure 2.

To understand whether the *NlRpn* and *NlRac* genes are involved in lipid metabolism in female ovaries and fat bodies, we investigated the effects of *NlRpn* and *NlRac* knockdown on *lipase* gene expression using qRT-PCR. Transcript levels of *adipose triglyceride lipase* (*NlATGL*), *pancreatic triacylglycerol lipase 1* (*NlPNLIP1*), *lipoprotein lipase-like 2* (*NlLPL2*) and *gastric triacylglycerol lipase-like 1* (*NlLIPF1*) genes in the ovaries were markedly decreased by knockdown of *NlRpn1–3*, *NlRpn6–8*, *NlRpn11–12* and *NlRpn14* compared with dsGFP-injected controls (figure 7*a*). Knockdown with ds*NlRpn4*, ds*NlRac5*, ds*NlRac2*, ds*NlRac1* and ds*NlRpn13* notably reduced the transcript levels of *NlATGL*, but had no effect on transcript levels of *NlPNLIP1*. Ds*NlRpn4*, ds*NlRac1* and ds*NlRpn13* significantly decreased the transcript levels of *NlLPL2* and *NlLIPF1*, whereas ds*NlRac5* and ds*NlRac2* had no effect on the transcript levels of these two genes. These data suggest that knockdown of *NlRpn* and *NlRac* genes affects lipid-metabolizing gene transcription in female ovaries.

In fat bodies, *NlATGL*, *pancreatic lipase-related protein 2* (*NlPLRP2*), and *pancreatic triacylglycerol lipase 2* (*NlPNLIP2*) genes displayed significant changes at the mRNA level after knockdown of *NlRpn*s. Ds*NlRpn1*-*3*, ds*NlRpn6–8*, ds*NlRpn11–12* and ds*NlRpn14* strikingly decreased *NlATGL*, *NlPLRP2* and *NlPNLIP2* transcript levels, while ds*NlRpn4*, ds*NlRac5*, ds*NlRac2*, ds*NlRac1* and ds*NlRpn13* had no effects on transcript levels compared with dsGFP-injected controls (figure 7*b*), implying that most *NlRpn* (*NlRpn1–3*, ds*NlRpn6–8*, ds*NlRpn11–12* and ds*NlRpn14*) genes are involved in the lipid metabolism in fat bodies.

### 2.8. Effect of RNA interference on expression of *NlVg* and *NlVg-like* genes in ovaries and fat bodies

**Figure 8.**
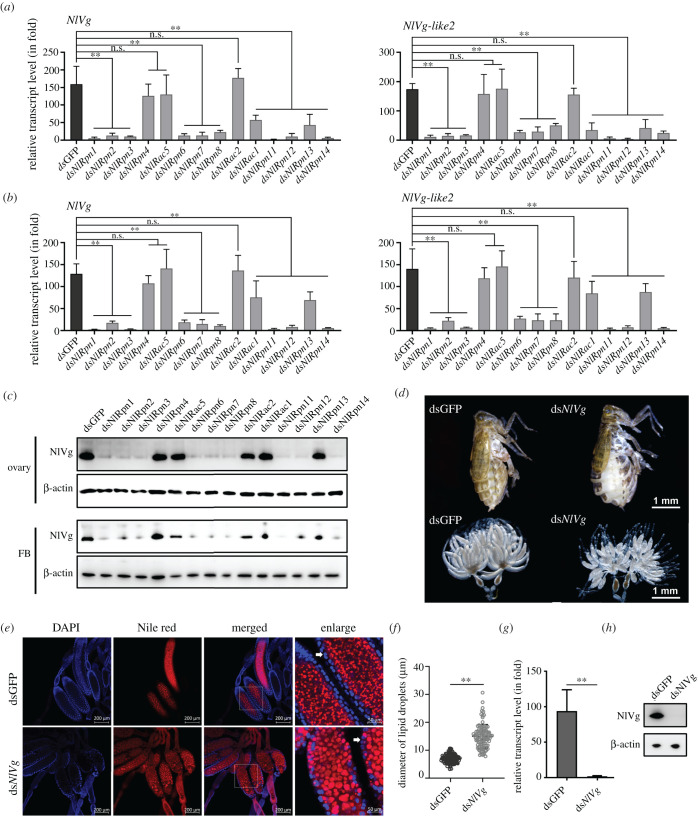
Effects of RNAi on *NlVg* and *NlVg-like* expression in ovaries and fat bodies. Transcript levels (in fold) of *NlVg* and *NlVg-like* genes after knockdown of *NlRpn*s and *NlRac*s in ovaries (*a*) and fat bodies (*b*) from ds*NlRpn*- or ds*NlRac*-injected females on the 5th day after emergence. dsGFP-injected samples were used as controls. Relative transcript levels of *NlVg* and *NlVg-like* genes in each treatment were determined by qRT-PCR. Three independent biological replicates (mean ± standard deviation; *n* = 10) were conducted, as described in figure 2. (*c*) Determination of NlVg protein levels in female ovaries and fat bodies from ds*NlRpn*- or ds*NlRac*-injected females on the 5th day after emergence, determined by western blot analysis. β-Actin was used as a loading control. (*d*) Ovarian and whole-body morphologies were observed under a stereomicroscope in ds*NlVg*-injected adult females on the 5th and 7th days after emergence. Scale bars, 1.0 mm. (*e*) Observation of lipid droplets in oocytes after knockdown of *NlVg*. Ovaries were isolated from ds*NlVg*-injected adult females on the 5th day after emergence and lipid droplets were observed as described in figure 5. (*f*) Lipid droplets were measured using ImageJ 1.52a software (National Institutes of Health, Maryland, USA). Statistical analysis was performed using GraphPad Prism 8. Lipid droplets in oocytes from ds*NlVg*- and dsGFP-injected females exhibited different diameters (mean ± standard deviation; *n* = 100).**p < 0.01 between ds*NlVg* and dsGFP treatments (Student's *t*-test). (*g*) *NlVg* gene transcript levels were analysed by qRT-PCR, as described in figure 2. (*h*) NlVg protein levels in ovaries from ds*NlVg*- and dsGFP-injected females on the 5th day after emergence, detected by western blot analysis (*n* = 10). Protein samples were diluted 400 times. β-actin was used as a loading control. Western blotting data represent one of three experiments.

Vg is the key factor in vitellogenesis in insects. We therefore investigated the effects of *NlRpn* and *NlRac* knockdown on expression levels of *NlVg* and *NlVg-like* gene transcription in female ovaries and fat bodies. qRT-PCR analysis indicated that ds*NlRpn*s (except ds*NlRpn4*) and ds*NlRac1* significantly downregulated *NlVg* and *NlVg-like2* transcripts in both ovaries and fat bodies (figure 8*a,b*). Western blotting assay using an antibody against *N. lugens* Vg antigen prepared in our laboratory further confirmed these results at the protein level. Faint-specific protein bands were detected in the ovaries and fat bodies of ds*NlRpn1–3*, ds*NlRpn6–8*, ds*NlRpn11–12* and ds*NlRpn14*-injected females, and stronger bands were detected in the ovaries of ds*NlRpn4*, ds*NlRac5*, ds*NlRac2*, ds*NlRac1* and ds*NlRpn13*-injected females (figure 8*c*). These findings indicate that the changes in *NlRpn*-mediated *Vg* expression patterns were consistent with that of *lipase* genes. We subsequently explored the physiological function of the *Vg* gene in lipid metabolism by RNAi. Knockdown of *Vg* in adult females generated deficient phenotypes, with abdominal hypertrophy and stretched intersegmental membranes in the tergum, and abnormal oocytes in the ovarioles (figure 8*d*). Lipid droplets in the oocytes were much larger in ds*Vg*-injected females (15 µm), compared with dsGFP-injected controls (6.99 µm) (figure 8*e,f*), suggesting remodelling of lipid droplets in the oocytes. Knockdown of *Vg* significantly decreased Vg levels in female ovaries at the 5th day after emergence at both the transcript (figure 8*g*) and protein levels (figure 8*h*), compared with dsGFP-injected controls. These investigations indicate that knockdown of *NlRpn1–3*, *NlRpn6–8*, *NlRpn11–12* and *NlRpn14* transcript levels had effects on *Vg* expression in female ovaries and fat bodies. To understand whether knockdown of *NlRpn*s affect other ovary- or oocyte-specific gene expression, we examined the transcript level variations of a *Vg receptor* (*VgR*) gene, which is specifically expressed in the ovary of *N. lugens* female, by knockdown of individual *NlRpn* or *NlRac* expression in *N. lugens*. No significant expression variations of the *NlVgR* gene were detected in the ovaries after knockdown of specific *NlRpn* or *NlRac* genes (electronic supplementary material, figure S1). These data suggest that *NlRpn*s affect the expression of the *NlVg* gene but did not affect the expression of the *NlVgR* gene in female ovaries.

### 2.9. Effect of *NlRpn* and *NlRac* knockdown on ovulation and egg hatching

**Figure 9.**
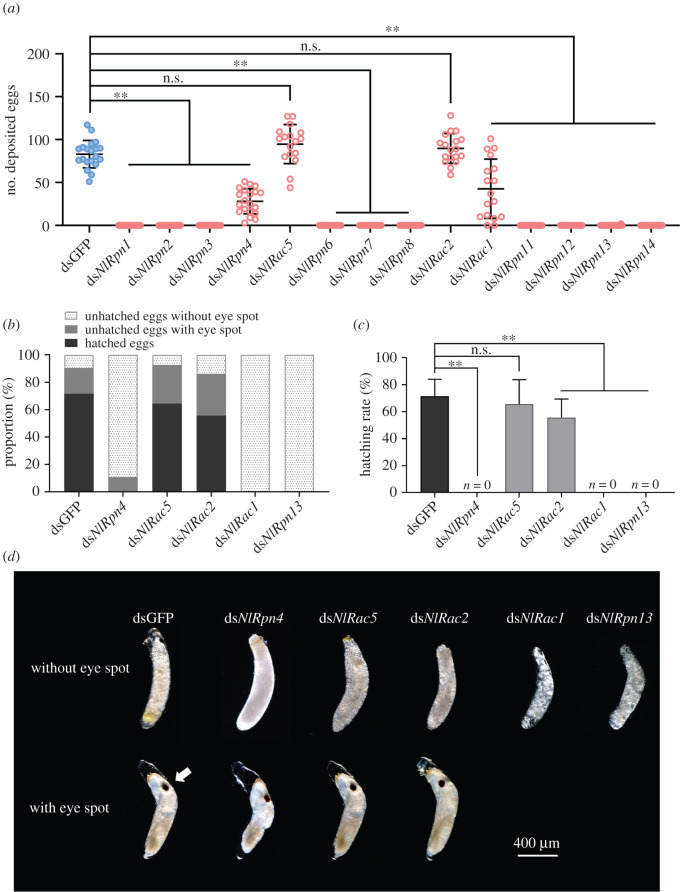
Effects of RNAi on female reproduction in *N. lugens*. (*a*) Number of laid eggs after knockdown of each *NlRpn* or *NlRac*. **p < 0.01 between indicated treatments (Student's *t*-test); ns: no significant difference. ds*NlRpn1*–*4* (*n* = 20), ds*NlRac5* (*n* = 17), ds*NlRpn6*–*8* (*n* = 20), ds*NlRac2* (*n* = 18), ds*NlRac1* (*n* = 17), and ds*NlRpn11*–*14* (*n* = 20). Blue and red dots indicate dsGFP and ds*NlRpn* or ds*NlRac* treatments, respectively. (*b*) Proportions of eggs with and without an eye spot. Eggs were allowed to hatch for 11 days and unhatched eggs were collected to calculate the proportions with and without an eye spot. (*c*) Hatching rates of eggs laid by ds*NlRpn*- or ds*NlRac*-injected females mated with non-injected males. Three biological replicates were carried out for each mating (*n* = 17–20). (*d*) Morphology of laid eggs. Eggs without an eye spot, which did not hatch into nymphs, are shown in the upper panel. Eggs with an eye spot are shown in the lower panel. Arrowhead indicates eye spot. Scale bar, 0.4 mm.

To further understand the functional roles of *NlRpn* and *NlRac* genes in reproduction, we investigated ovulation in adult females and hatchability of the eggs following *NlRpn* and *NlRac* knockdown in newly emerged adult females. Following mating with non-injected males, females that were treated with ds*NlRpn1*, ds*NlRpn2*, ds*NlRpn3*, ds*NlRpn6*, ds*NlRpn7*, ds*NlRpn8*, ds*NlRpn11*, ds*NlRpn12* or ds*NlRpn14* failed to lay eggs, suggesting that these genes were required for oocyte maturation and ovulation (figure 9*a*). Furthermore, the average numbers of eggs laid by ds*NlRpn4-*, ds*NlRac1-* and ds*NlRpn13*-injected females were significantly lower than those laid by dsGFP-injected controls (figure 9*a*). Ds*NlRpn4-* and ds*NlRac1*-injected females laid an average of 28 and 43 eggs, respectively, while ds*NlRpn13*-injected females laid an average of 0.1 eggs. By contrast, ds*NlRac5-* and ds*NlRac2*-injected females laid 95 and 90 eggs, respectively, similar to dsGFP-injected females (83 eggs). Furthermore, ds*NlRac1*- and ds*NlRpn13*-injected females laid eggs without an eye spot (eye pigmentation) (figure 9*b*), ds*NlRpn4*-injected females laid 89% of eggs without and 11% with an eye spot, and ds*NlRac5*- and ds*NlRac2*-injected females laid 93% and 86% of eggs with an eye spot, similar to dsGFP-injected controls (90%). In *N. lugens*, an eye spot usually appears 4–5 days after ovulation, and eggs with an eye spot are considered viable, whereas those without an eyespot are considered to be dead [50]. None of the eggs laid by ds*NlRpn4-*, ds*NlRac1-* or ds*NlRpn13*-injected females hatched into nymphs (figure 9*c*), compared with around 66% of eggs laid by ds*NlRac5*-injected females, which was similar to the 72% hatching rate in dsGFP-injected controls. Approximately 56% of the eggs laid by ds*NlRac2*-injected females developed into nymphs, which was significantly lower than in dsGFP-injected controls (figure 9*c*). Eggs with and without eye spots laid on rice leaf sheaths by ds*NlRpn4-*, ds*NlRac5-*, ds*NlRac2-*, ds*NlRac1-*, ds*NlRpn13-* and dsGFP-injected females are shown in figure 9*d*.

### 2.10. Effects of RNA interference on *halloween* gene expression and 20E synthesis in adult female *Nilaparvata lugens*

**Figure 10.**
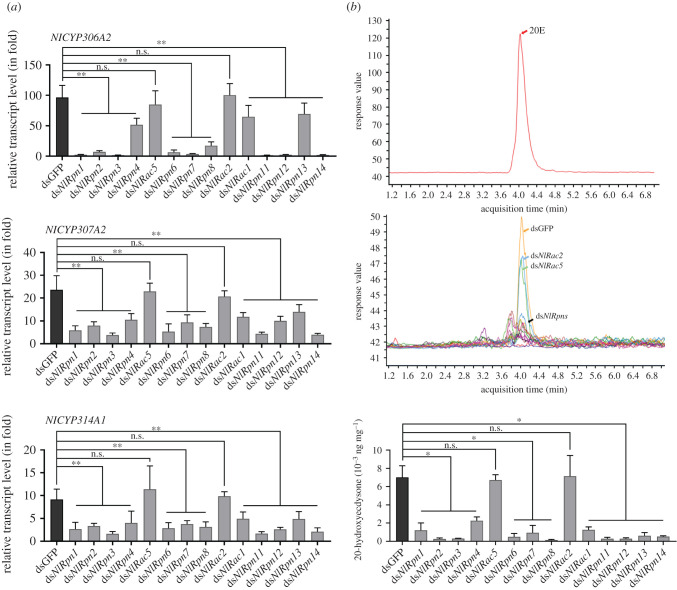
Effect of RNAi on 20E biosynthesis in adult female *N. lugens*. (*a*) Transcript levels of 20E biosynthetic enzyme genes in ovaries of ds*NlRpn*- or ds*NlRac*-injected females on the 5th day after emergence. dsGFP-injected samples were used as controls. Relative transcript levels of the target genes in each treatment were determined by qRT-PCR. Three independent biological replicates (mean ± standard deviation; *n* = 10) were conducted, as described in figure 2. (*b*) 20E levels in whole bodies of ds*NlRpn*- and ds*NlRac*-injected adult females at 120 h after emergence, determined by LC-MS/MS. Insects injected with dsGFP were used as controls. Chromatograms of standard 20E solution (upper) and 20E extracted from ds*NlRpn*- and ds*NlRac*-injected adult females (lower). Orange, blue, and green arrows indicate dsGFP, ds*NlRac2* and ds*NlRac5* treatments, respectively; black arrow indicates all other ds*NlRpn* and ds*NlRac* treatments. (*c*) Quantification of 20E in adult females at 120 h after emergence. 20E levels in each ds*NlRpn* or ds*NlRac* treatment represent the mean ± standard deviation per microgram of whole bodies in three independent experiments. For each independent experiment, approximately 20 adult females (each total 50 mg) were used to quantify 20E amounts, as described in Zhou *et al.* [45].

We further investigated the effect of RNAi on the expression of genes encoding 20E biosynthetic enzymes by qRT-PCR analysis targeting *halloween* genes, including *NlCYP307A2*, *NlCYP307B1*, *NlCYP306A2*, *NlCYP302A1*, *NlCYP315A1* and *NlCYP314A1*. Transcript levels of *NlCYP307A2*, *NlCYP306A2* and *NlCYP314A1* were significantly decreased after knockdown of *NlRpn*s and *NlRac1*, but were not affected by knockdown of *NlRac5* and *NlRac2* (figure 10*a*), indicating that *NlRpn*s and *NlRac1* mediated 20E biosynthetic gene expression. We previously quantified 20E levels in 4th–5th instar nymphs and adults at 0*–*72 h after emergence [45]. In the present study, we quantified 20E levels in females at 120 h after emergence, as the oocytes matured in the ovaries. A typical chromatogram of 20E was produced from dsGFP-injected adult females at 120 h using liquid chromatography–tandem mass spectrometry (LC-MS/MS). The MS/MS spectra of 20E extracted from ds*NlRac5*- and ds*NlRac2*-injected females were identical to the typical 20E spectra (figure 10*b*), but spectra from other ds*NlRpn*- and ds*NlRac1*-injected females produced very small 20E peaks (figure 10*b*), suggesting that ds*NlRpn* and ds*NlRac1* RNAi significantly reduced 20E levels in adult female *N. lugens*. Furthermore, we quantified the amounts of 20E in adult females at 120 h after emergence (figure 10*c*). 20E levels were very low in ds*NlRpn*- and ds*NlRac1*-injected adult females (0.0001–0.002 ng mg^−1^) compared with dsGFP-, ds*NlRac5-* and ds*NlRac2*-injected females (0.0067–0.0072 ng mg^−1^). These data suggest that knockdown of *NlRpn*s and *NlRac1* affects transcription of 20E biosynthetic enzymes and production of 20E in female adults. We conducted rescue experiments by injecting 0.4 µg of 20E in each ds*NlRpn*-injected insect. The phenotype of female ovaries was observed at 120 h after emergence. 20E could not rescue the abnormal ovary phenotypes resulting from ds*NlRpn* treatments (data not shown).

### 2.11. Effects of proteasome activity on ovarian morphology and gene transcription in females

**Figure 11.**
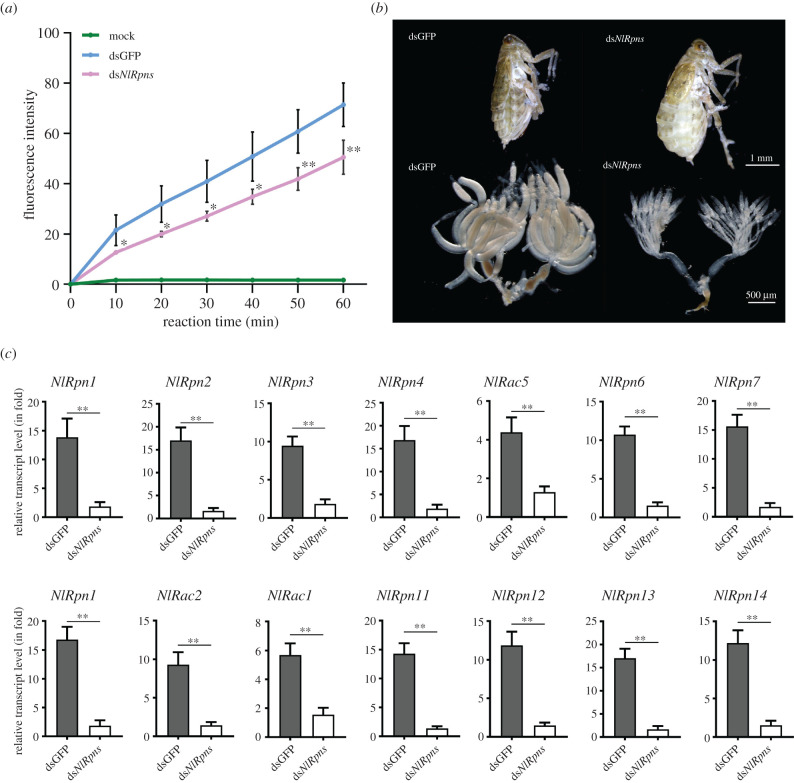
RNAi effects of a mixture of ds*NlRpn*s and ds*NlRac*s on ovarian development. Newly emerged adult females were injected with a mixture of ds*NlRpn*s and ds*NlRac*s including ds*NlRpn*1-4, ds*NlRac5*, ds*NlRpn6*-*8*, ds*NlRac2*, ds*NlRac1* and ds*NlRpn11*-*14* at an equal quality ratio. (*a*) Measurement of chymotrypsin-like proteasome activity in the whole-body of the adult females using the fluorogenic peptide Suc-Leu-Leu-Val-Tyr-AMC. *y*-axis refers to the fluorescence intensities measured at 10 min intervals in ds*NlRpn* + *NlRac* mix- and dsGFP-injected samples. *x*-axis refers to the reaction time over a period of test time (60 min). Each sample represents 20 mg of the whole-body lysates of the adult females. For each treatment, five independent biological replicates were conducted. **p < 0.01, *p < 0.05 between ds*NlRpn* + *NlRac* mix- and dsGFP treatments (Student's *t*-test). Data are means ± standard deviation. Statistical analysis was performed using GraphPad Prism 8. (*b*) Morphology of adult females was observed on the 7th day after RNAi. Scale bar, 1 mm. Ovaries were dissected from the females on the 5th day after RNAi. Insects injected with dsGFP were used as controls. Scale bar, 500 µm. (*c*) RNAi effects depleting *NlRpn*s and *NlRac*s together on individual *NlRpn* and *NlRac* gene expression. RNAi was performed by microinjecting ds*NlRpn* + *NlRac* mix into the newly emerged female adults. Total RNAs were extracted from the whole bodies of the insects on the 5th day after RNAi (*n* = 10 adults). The relative transcript levels of individual *NlRpn* and *NlRac* gene were determined by qRT-PCR as described in figure 2. Statistical analysis was performed using GraphPad Prism 8 software. Three independent biological replicates (mean ± standard deviation) were conducted and the relative transcript levels were measured using the ΔΔ*C_t_* method. **p < 0.01 between dsGFP and ds*NlRpn* + *NlRac* mix treatments (Student's *t*-test). ds*NlRpn*s refers to ds*NlRpn* + *NlRac* mix.

As described above, knockdown of *NlRpn*s resulted in various female reproduction phenotypes. To explore the possibility that these phenotypes resulted from decreases or changes in proteolysis within *N. lugens*, we determined the chymotrypsin-like proteasome activity through *in vitro* proteasome activity assay after knockdown of transcript levels of all *NlRpn*s and *NlRac*s including *NlRpn1–4*, *NlRpn6–8*, *NlRpn11–14*, *NlRac5*, *NlRac2* and *NlRac1* genes using microinjection of a mixture of ds*NlRpn*s and ds*NlRac*s into the newly emerged females. As shown in figure 11, knockdown of these genes significantly decreased the chymotrypsin-like proteasome activity in adult females when compared to dsGFP-injected female samples over a period of 60 min (figure 11*a*). The fluorescence intensities in ds*NlRpn*s and ds*NlRac*s-injected female samples were 1.4*–*1.7 fold lower than that in dsGFP-injected female samples within 60 min, indicating that *NlRpn* and *NlRac* knockdown reduced proteasome activity in adult females. The knockdown of *NlRpn*s and *NlRac*s mixture generated deficient ovaries in the adult females (figure 11*b*). Simultaneous ds*NlRpn*s and ds*NlRac*s knockdown significantly decreased individual *NlRpn* or *NlRac* transcript levels in the adult female (figure 11*c*).

**Figure 12.**
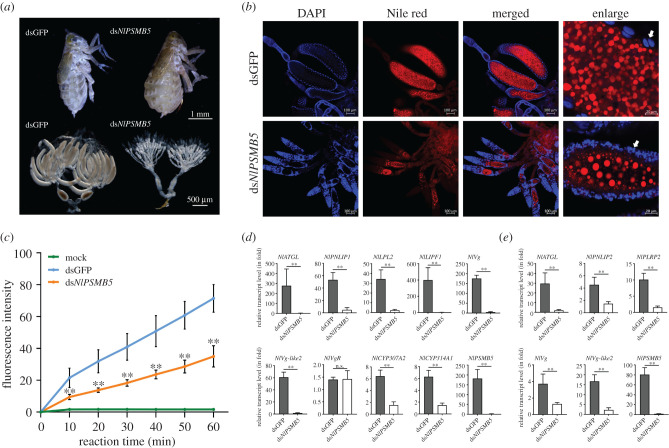
Effects of *NlPSMB5* RNAi on ovarian development and lipid accumulation in oocytes. (*a*) The whole-body and ovarian morphologies were observed under a stereomicroscope in ds*NlPSMB5*-injected adult females on the 5th day after RNAi. Scale bars, 1.0 mm and 500 µm, respectively. (*b*) Observation of lipid droplets in oocytes after knockdown of *NlPSMB5*. Ovaries were isolated from ds*NlPSMB5*-injected adult females on the 5th day after RNAi and lipid droplets were observed as described in figure 6. White arrows indicate the nuclei of the follicular cells. (*c*) Chymotrypsin-like proteasome activity of the whole-body lysates was measured using Suc-Leu-Leu-Val-Tyr-AMC. *y*-axis refers to the fluorescence intensities measured at 10 min intervals in ds*NlPSMB5*- and dsGFP-injected samples. Mock refers to a sample without addition of the supernatants from the whole-body lysates and used as a negative control. *x*-axis refers to the reaction time over a period of test time (60 min). Each sample represents 20 mg of the whole-body lysates of adult females. For each treatment, five independent biological replicates were conducted. **p < 0.01 between ds*NlPSMB5* and dsGFP treatments (Student's *t*-test). Data are means ± standard deviation. Statistical analysis was performed using GraphPad Prism 8. The effects of *NlPSMB5* knockdown on the transcript level variations of *Vg*, *VgR*, *lipase* and *halloween* genes. Transcript levels of the genes were determined in ovary (*d*) and fat body (*e*) of ds*NlPSMB5*-injected adult females on the 5th day after RNAi. dsGFP-injected samples were used as controls. Transcript level variations of the target genes were determined by qRT-PCR. Three independent biological replicates (mean ± standard deviation; *n* = 10) were conducted, as described in figure 2.

Subsequently, we investigated the effects of decreasing proteolysis through knockdown of a subunit in the 20S CP by RNAi. Subunits *β*2, *β*5 and *β*1 of the 20S CP have proteolytic activity, displaying trypsin-like, chymotrypsin-like and caspase-like peptidase activity, respectively. Here, we focus on a *N. lugens proteasome subunit β5* (*NlPSMB5*) gene to examine RNAi effects on ovarian morphology and lipid accumulation in oocytes. Knockdown of *NlPSMB5* generated an abnormally inflated abdomen and stretched intersegmental membranes in the tergum of adult females (figure 12*a*, upper panel). These females displayed apparently abnormal ovaries with no or malformed immature oocytes in the ovarioles (figure 12*a*, lower panel). Globular lipid droplets with different sizes were observed in the immature oocytes (figure 12*b*). By contrast, dsGFP-injected females had normal ovaries containing the mature oocytes with the uniform sizes of lipid droplets (figure 12*a*,*b*). We conducted a kinetic assay to monitor the chymotrypsin-like proteasome activity in adult females by knockdown *NlPSMB5* gene. Our experiments revealed that *NlPSMB5* knockdown significantly decreased the chymotrypsin-like proteasome activity in adult females over a period of 60 min. In dsGFP-injected female samples, the fluorescence intensities were 2.0*–*2.3 fold higher than that in ds*NlPSMB5*-injected sample within 60 min (figure 12*c*). A negative control did not show the change of the fluorescence intensities over the test period. We investigated the effects of *NlPSMB5* knockdown on the transcript level variations of *Vg*, *VgR* and *lipase* genes in fat bodies and ovaries of adult females. qRT-PCR analysis confirmed that transcript levels of *NlPSMB5* gene were notably reduced in ds*NlPSMB5*-injected fat bodies and ovaries compared with those in dsGFP-injected controls (figure 12*d*). The transcript levels of the *NlATGL*, *NlPNLIP1*, *NlLPL2*, *NlLIPF1*, *NlVg*, *NlVg-like2*, *NlCYP307A2* and *NlCYP314A1* genes were significantly decreased in the ovaries (figure 12*d*); and the transcript levels of *NlATGL*, *NlPNLIP2*, *NlPLRP2*, *NlVg* and *NlVg-like2* genes were significantly decreased in the fat bodies by knockdown of *NlPSMB5* compared with dsGFP-injected controls (figure 12*e*). By contrast, *NlPSMB5* knockdown did not change the *NlVgR* transcript levels in the ovaries (figure 12*d*). These observations suggest that decreasing chymotrypsin-like proteasome activity through decreasing a subunit in the 20S CP by RNAi resulted in decreases of transcription of the key factors critical for female reproduction in *N. lugens*.

### 2.12. Effects of oocyte-specific *NlBic-C* knockdown on gene expression in female ovaries

**Figure 13.**
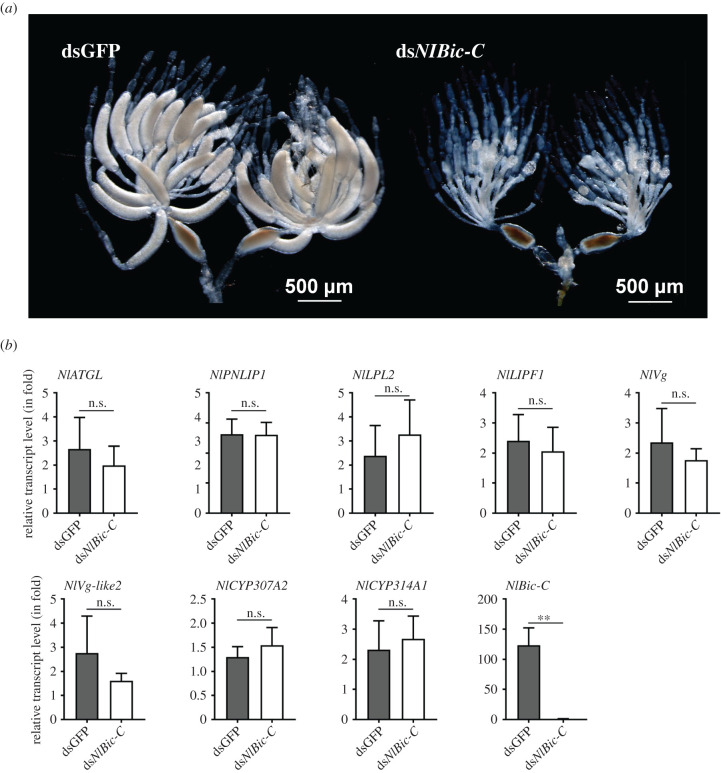
RNAi effects of ds*NlBic-C* on ovarian development. Newly emerged adult females were injected with ds*NlBic-C*. (*a*) Ovaries were dissected from the females on the 5th day after RNAi. Insects injected with dsGFP were used as controls. Scale bar, 500 µm. (*b*) RNAi effects depleting *NlBic-C* on the transcript levels of lipid-metabolizing (*NlATGL*, *NlPNLIP1*, *NlLPL2* and *NlLIPF1*), 20E biosynthetic enzymes (*NlCYP307A2*, *NlCYP306A2* and *NlCYP314A1*) and *Vg* (*NlVg* and *NlVg-like2*) genes. RNAi was performed by microinjecting ds*NlBic-C* into the newly emerged female adults. Total RNAs were extracted from the ovaries of the females on the 5th day after RNAi (*n* = 10 adults). The relative transcript levels of the target genes were determined by qRT-PCR as described in figure 2. Statistical analysis was performed using GraphPad Prism 8 software. Three independent biological replicates (mean ± standard deviation) were conducted and the relative transcript levels were measured using the ΔΔ*C_t_* method. **p < 0.01 between dsGFP and ds*NlBic-C* treatments (Student's *t*-test). ns: no significant difference.

As *NlRpn* knockdown decreased the levels of lipid-metabolizing, 20E biosynthetic enzymes, *Vg* and *Vg*-like, and halted oogenesis at late mid-stage, we investigated whether the levels of these genes could be altered after knockdown of genes encoding non-proteasomal subunits that are also known to affect oogenesis. We used female-specific *N. lugens Bicaudal-C* (*NlBic-C*) as a control because this gene is expressed only in developing oocytes and required for oogenesis and oocyte maturation. *NlBic-C* knockdown resulted in abnormal ovaries with severely inhibited oocyte growth in ovarioles (figure 13*a*), whereas fully developed oocytes were observed in the ovarioles of dsGFP-injected female ovaries (figure 13*a*). Subsequently, we investigated the effects of *NlBic-C* knockdown on *Vg* and *Vg*-like levels. qRT-PCR analysis showed that *NlBic-C* expression was significantly decreased on the 5th day after emergence in ds*NlBic-C*-injected females (figure 13*b*). *NlBic-C* knockdown did not significantly alter the expression of lipid-metabolizing genes (*NlATGL*, *NlPNLIP1*, *NlLPL2* and *NlLIPF1*), 20E biosynthetic enzymes (*NlCYP307A2* and *NlCYP314A1*) and *Vg* genes (*NlVg* and *NlVg*-*like2*) (figure 13*b*). These results indicate that knockdown of *NlBic-C*, which is necessary for the late stages of oogenesis, did not affect the levels of lipid-metabolizing genes, 20E biosynthetic enzymes and the *Vg* gene, whereas knockdown of *NlRpn*s affected the expression of these genes in adult females.

## 3. Discussion

Rpns are constituents of the 26S proteasome that have been reported to be involved in polyubiquitinated substrate protein recognition and deubiquitination in eukaryotes. Using RNAi, we report the functions of *Rpn* and *Rac* in *N. lugens*, a model hemipteran insect, based on the whole genome sequence. Fifteen *Rpn*s and *Rac*s were identified in the *N. lugens* genome and transcriptome databases, and their deduced amino acid sequences showed close phylogenetic relationships with their homologues in many insect species and humans. In *D. melanogaster*, however, the nomenclatures of *Rpn*s were different from those of other insect species and humans but similar to their homologues in *S. cerevisiae*. Bioinformatics analysis indicated that each NlRpn and NlRac contained a characteristic domain(s), including PC, PAM, PCI, vWFA, UIM, JAB/MPN, MitMem, PDZ and ANK, implying their functional diversity. qRT-PCR analysis detected *NlRpn* and *NlRac* expression throughout development, with the highest levels in females and/or laid eggs and the lowest levels in males, suggesting their functional importance in adult females. Tissue-specific expression analysis revealed that *NlRpn* and *NlRac* levels were highest in the ovaries, further supporting their physiological roles in female reproduction.

Based on these observations, we silenced *NlRpn* and *NlRac* expression in *N. lugens* by RNAi, which resulted in the generation of lethal phenotypes. The survival rates of insects injected with ds*NlRpn*s and ds*NlRac1* were less than 20% at 14 dpi compared with greater than 80% in dsGFP-injected controls, suggesting that these genes are important for survival. However, *NlRac5* and *NlRac2* were not required for survival. Except for *NlRpn4*, *NlRac5* and *NlRac2*, adult female *N. lugens* injected with ds*NlRpn*s and ds*NlRac*s showed an abnormally expanded abdomen and a significantly increased body weight at 7 dpi.

To determine whether knockdown of *NlRpn* or *NlRac* had any effect on other *NlRpn*s and *NlRac*s, we examined the levels of other *NlRpn*s and *NlRac*s in the absence of a different *NlRpn* or *NlRac*. Our results revealed that knockdown of *NlRpn* or *NlRac* either upregulated or failed to alter the levels of most other *NlRpn*s and *NlRac*s. In *C. elegans*, knockdown of *rpn*-*6.1* or *rpn*-*10* increased the expression of the other 26S proteasome subunits, probably to compensate for the reduction in proteasomal activity induced by the decreased levels of these subunits [36,37]. In this study, we report that knockdown of *NlRpn* or *NlRac* did not affect the levels of most other *NlRpn*s and *NlRac*s.

To understand the reasons for the abnormally expanded abdomen phenotype, we dissected adult females and observed fat bodies in the abdomens of individuals injected with most ds*NlRpn*s and ds*NlRac1*. However, we observed fewer fat bodies in ds*NlRpn4-*, ds*NlRac5-*, ds*NlRac2-* and dsGFP-injected females (data not shown). Ds*NlRpn1*-, ds*NlRpn2*-, ds*NlRpn3*-, ds*NlRpn6*-, ds*NlRpn7*-, ds*NlRpn8*-, ds*NlRac1*-, ds*NlRpn11*-, ds*NlRpn12*-, ds*NlRpn13*- and ds*NlRpn14*-injected females also showed defects in the ovaries, which contained no or irregularly shaped immature oocytes in the ovarioles. By contrast, ds*NlRpn4-*, ds*NlRac5-*, ds*NlRac2-* and dsGFP-injected females showed normal ovaries with mature banana-shaped oocytes. These findings indicate that most *NlRpn*s and *NlRac1* might be involved in lipid metabolism in the fat bodies, that is, they might affect the absorption and utilization of lipids in the developing oocytes. We therefore investigated lipid droplet accumulation in the ovary. Knockdown of *NlRpn*s resulted in the accumulation of large lipid droplets in the oocytes, whereas ds*NlRpn4-*, ds*NlRac5-*, ds*NlRac2-* and dsGFP-injected females showed small round lipid droplets that were distributed uniformly in the oocytes.

In Hemipteran insects, such as *Cixius nervosus* (Cixiidae) and *Javesella pellucida*, *Conomelus anceps*, *N. lugens* (Delphacidae), the developing oocytes are surrounded by a single layer of follicular cells. At the start of vitellogenesis, each follicular cell generates two nuclei, with the two nuclei positioned one on top of the other in the long axis of each cell [16,51]. Knockdown of *NlRpn*s disrupted the arrangement of follicular cells surrounding the oocytes. Furthermore, these follicular cells did not possess the typical two nuclei, whereas ds*NlRpn4-*, ds*NlRac5-*, ds*NlRac2-* and dsGFP-injected females had normally arranged follicular cells with two nuclei surrounding the oocytes. These observations support the hypothesis that *NlRpn*s are associated with the accumulation of lipid droplets in oocytes and the development of follicular cells during vitellogenesis in female adults.

Lipids, mostly triglycerides, comprise approximately 30*–*40% of the oocyte dry weight and greater than 50% of the fat body dry weight in insects [52]. Triglycerides are stored in lipid droplets in oocytes and fat body cells and serve as the main energy source for oocyte maturation and embryonic development [53–55]. We measured the triglyceride content of the ovaries and fat bodies in *N. lugens*. Except for *NlRac5*, knockdown of *NlRpn*s and *NlRac*s significantly reduced triglyceride levels in the ovaries, but not in fat bodies. Combined with the abnormally expanded abdomens of *NlRpn*-injected females, these results indicate that lipid metabolism was adversely affected in the fat bodies, thereby disrupting the provision of triglycerides to the ovaries as well as the uptake and utilization of triglycerides in the oocytes.

We further investigated the reasons for the triglyceride metabolic disorder by examining *lipase* expression in the ovaries and fat bodies following RNAi. The levels of *NlATGL*, *NlPNLIP1* and *NlLIPF1*, which encode triglyceride metabolism-related lipases, and *NlLPL2*, which encodes a lipoprotein lipase, were significantly decreased in the ovaries, and the levels of *NlATGL* and *NlPNLIP2*, which encode triglyceride metabolism-related lipases, and *NlPLRP2*, which encodes a pancreatic lipase-related protein, were significantly decreased in the fat bodies after the knockdown of *NlRpn*s and *NlRac*s, but not *NlRpn4*, *NlRac5*, *NlRac2*, *NlRac1* and *NlRpn13*. These results indicate that knockdown of most *NlRpn*s can affect *lipase* expression in the ovary and fat bodies.

*ATGL* is a vital enzyme in the first step of triglyceride hydrolysis [55,56], and the suppression of *ATGL* expression significantly reduced the glycerin content and the number of eggs laid in *N. lugens* [57]. Another study has reported that a lipoprotein lipase is important in the uptake of lipids by developing oocytes in *Manduca sexta* [58]. *PLRP2* has various lipolytic activities in mammals, but its role in female reproduction remains unknown. We previously reported that *NlPLRP2* was required for oocyte maturation and development in insects [18]. The findings of the present study reveal the regulatory mechanisms of six genes encoding various lipases in *N. lugens*. The suppression of *lipase* expression by knockdown of *NlRpn*s resulted in triglyceride metabolism disorders, leading to lipid accumulation in the ovaries and fat bodies, abnormally expanded abdomens, and large lipid droplet accumulation in developing oocytes in affected females. The oocytes were probably unable to use the products of the lipase reaction, such as glycerol and free fatty acids, thereby impairing lipid hydrolysis metabolism and oocyte maturation.

Developing oocytes in all oviparous insects accumulate massive amounts of yolk to ensure embryo development [59]. Vg, as the major yolk protein precursor, is synthesized in fat bodies outside of the ovaries, released into the haemolymph, and transported into the growing oocytes via membrane receptor-mediated endocytosis [59,60]. The uptake of Vg by developing oocytes during egg maturation is essential for successful female reproduction [60]. *Vg* knockdown resulted in ovarian atrophy and reduced egg production in the hemipteran bedbug *Cimes lectularius* [61]. One *Vg* and two *Vg-like* (*Vg-like1* and *Vg-like2*) genes were identified and investigated in oocyte development in *N. lugens* [3]. In the present study, we examined the changes in the levels of these genes following *NlRpn* and *NlRac* knockdown. Except for *NlRpn4*, *NlRac5*, *NlRac2*, *NlRac1* and *NlRpn13*, knockdown of *NlRpn*s and *NlRac*s significantly decreased the levels of *Vg* and *Vg*-*like2*, but not *Vg-like1*, in the ovaries and fat bodies, as confirmed at the protein level by western blotting. These data suggest that *Vg* and *Vg*-*like2* expression is affected by knockdown of most *NlRpn*s. Furthermore, the suppression of *Vg* expression in adult females resulted in the accumulation of hypertrophied fat bodies in the abnormally expanded abdomen and large lipid droplets in the developing oocytes, consistent with the observations of *NlRpn* gene suppression. However, *Vg-like2* knockdown did not produce an abnormal phenotype in the oocytes and ovaries. Vg and Vg-like2 shared a close phylogenetic relationship that was distant from Vg-like1 in *N. lugens* [3]. In addition, *Vg* and *Vg*-*like2* shared the same developmental stage-specific expression patterns, with transcripts primarily detected in adults, whereas *Vg-like1* transcripts were detected throughout all stages, implying different regulatory mechanisms and physiological functions in *N. lugens* development and reproduction. In the present study, *NlRpn*s affected *Vg* and *Vg*-*like2* expression, and Vg accumulation in the ovaries and fat bodies. Vg is a primary yolk protein that provides nutrients for the oocytes, whereas Vg-like2 is not essential for oocyte maturation. Vg is taken up by developing oocytes via VgR-mediated endocytosis. qRT-PCR analysis indicated that knockdown of *NlRpn*s or *NlRac*s did not alter the levels of *NlVgR* in the ovaries, suggesting that the depletion of *NlRpn*s or *NlRa*cs did not affect the uptake of Vg into developing oocytes. Instead, it impeded Vg production in ovaries and fat bodies, thereby disrupting oocyte maturation.

Except for *NlRac5* and *NlRac2*, knockdown of *NlRpn*s and *NlRac*s resulted in the complete failure of ovulation or a significant reduction in the number of eggs laid, which was owing to the severely deformed ovaries. The eggs laid by *NlRpn4*-, *NlRac1*- and *NlRpn13*-injected females failed to hatch into nymphs, and all of the eggs laid by ds*NlRac1*- and ds*NlRpn13*-injected females, and nearly 90% of those laid by ds*NlRpn4*-injected females, lacked an eye spot. These observations indicate that knockdown of *NlRpn*s can downregulate *lipase* and *Vg* expression, which might impede the digestion and absorption of nutrients and energy in the developing oocytes, thereby inhibiting oocyte development.

Ecdysteroids are steroid hormones in insects that are required for ovarian development and egg production. Ecdysteroids are produced in the ovaries of adult female insects [62,63]. We previously reported that 20E was an active steroid hormone in *N. lugens*, and knockdown of 20E biosynthetic enzymes in newly emerged females resulted in failed egg production, fewer vitellogenic mature oocytes, fewer eggs laid, and abnormal embryonic development of the laid eggs, demonstrating that 20E biosynthesis in ovaries is critical for ovarian development in *N. lugens* [45]. In the present study, we investigated the changes in expression of *halloween* genes in the 20E biosynthetic pathway in *N. lugens* ovaries following knockdown of *NlRpn*s and *NlRac*s. Except for *NlRac5* and *NlRac2*, knockdown of *NlRpn*s and *NlRac*s significantly decreased the levels of the *halloween* genes *NlCYP306A2*, *NlCYP307A2* and *NlCYP314A1* in female ovaries and reduced 20E production throughout the whole body in adult females. Among the *halloween* genes in *N. lugens*, *NlCYP306A2*, *NlCYP307A2* and *NlCYP314A1* were highly expressed in the ovaries, whereas *NlCYP307B1* and *NlCYP315A1* were expressed in various tissues [45], indicating that *NlRpn*s are involved in the expression of ovarian-specific *halloween* genes. *NlRpn*-mediated *halloween* gene knockdown resulted in reproductive failure. We conducted rescue experiments in ds*NlRpn*-injected insects and observed that 20E could not rescue the abnormal ovarian phenotype. This might have been owing to the fact that knockdown of *NlRpn*s decreased the expression of many genes, including *Vg*, *lipases*, and 20E biosynthetic genes, which are vital for oocyte maturation and development. The biosynthesis of 20E alone was not enough to rescue the reproductive defects.

The roles of *NlRpn*s and *NlRac*s in *N. lugens* survival and reproduction are summarized in table 2. Among the *NlRpn*s, *NlRpn1*-*3*, *NlRpn6*-*8*, *NlRpn11*-*12* and *NlRpn14* are associated with the expression of functional genes encoding a Vg and six lipases, including four triglyceride metabolism-related *lipases* and three *halloween* genes in the ovaries and/or fat bodies, which are necessary for oocyte formation and maturation. *NlRpn4*, *NlRpn13*, and an associated chaperone, *NlRac1*, are also involved in *Vg*, *lipase*, and *halloween* expression, and affect oocyte maturation and egg hatching. By contrast, two associated chaperones, *NlRac5* and *NlRac2*, had no effect on *Vg*, *lipase* and *halloween* expression. In addition, they had no effect on female development and reproduction, including oocyte maturation, ovulation, and egg hatching.

**Table 2.** Effects of RNAi on survival and reproduction in *N. lugens.* (Y, deficient phenotype; N, no deficient phenotype.)

target genes	deficient phenotypes			
	lethality	oocyte maturation	ovulation	hatching
*NlRpn1*	Y	Y	Y	Y
*NlRpn2*	Y	Y	Y	Y
*NlRpn3*	Y	Y	Y	Y
*NlRpn4*	Y	N	Y	Y
*NlRac5*	N	N	N	N
*NlRpn6*	Y	Y	Y	Y
*NlRpn7*	Y	Y	Y	Y
*NlRpn8*	Y	Y	Y	Y
*NlRac2*	N	N	N	Y
*NlRac1*	Y	Y	Y	Y
*NlRpn11*	Y	Y	Y	Y
*NlRpn12*	Y	Y	Y	Y
*NlRpn13*	Y	Y	Y	Y
*NlRpn14*	Y	Y	Y	Y

It is well established that the proteasome initiates gene transcription through proteolytic and non-proteolytic activities, and an inhibition of 26S proteasome function can affect transcription [46,47]. To determine whether the transcriptional changes reported in the present study were owing to a decrease in proteolytic activity or the functions of *NlRpn*s and *NlRac*s, we examined the effects of decreasing proteolysis by silencing the *NlPSMB5* subunit in the 20S CP. *NlPSMB5* knockdown notably decreased chymotrypsin-like proteasome activity in *N. lugens*, significantly reduced the levels of *NlATGL*, *NlPNLIP1*, *NlLPL2*, *NlLIPF1*, *NlVg*, *NlVg-like2*, *NlCYP307A2* and *NlCYP314A1* but not that of the ovarian-specific *VgR* gene, and generated deficient phenotypes in the ovaries of adult females, as observed in ds*NlRpn*-injected females, suggesting that the changes were owing to a decrease in proteolytic activity. We also investigated the effects on proteolytic activity following simultaneous ds*NlRpn*s and ds*NlRac*s knockdown. Knockdown significantly decreased chymotrypsin-like proteasome activity in adult females and resulted in a deficient phenotype in the ovaries. These observations indicate that depleting *NlRpn* decreased proteasome activity, which downregulated the transcription of genes involved in ecdysteroidogenesis, vitellogenesis and lipogenesis, and resulted in ovaries lacking the late stages of oogenesis.

To further confirm the results, we included a developing oocyte-specific gene, *NlBic-C*, whose knockdown halted oogenesis as a control to determine whether the same transcripts could be affected under non-Rpn knockdown conditions. qRT-PCR analysis showed that *NlBic-C* knockdown did not alter the levels of lipid-metabolizing genes (*NlATGL*, *NlPNLIP1*, *NlLPL2* and *NlLIPF1*), 20E biosynthetic enzymes (*NlCYP307A2* and *NlCYP314A1*), and *Vg* genes (*NlVg* and *NlVg*-*like2*) in female ovaries. By contrast, the levels of these genes were significantly decreased in *NlRpn*s or *NlPSMB5*-depleted ovaries compared with dsGFP-injected controls. These results indicate that *NlRpn* or *NlPSMB5* knockdown decreases proteasome activity and affects the same genes involved in ecdysteroidogenesis, vitellogenesis and lipogenesis.

**Figure 14.**
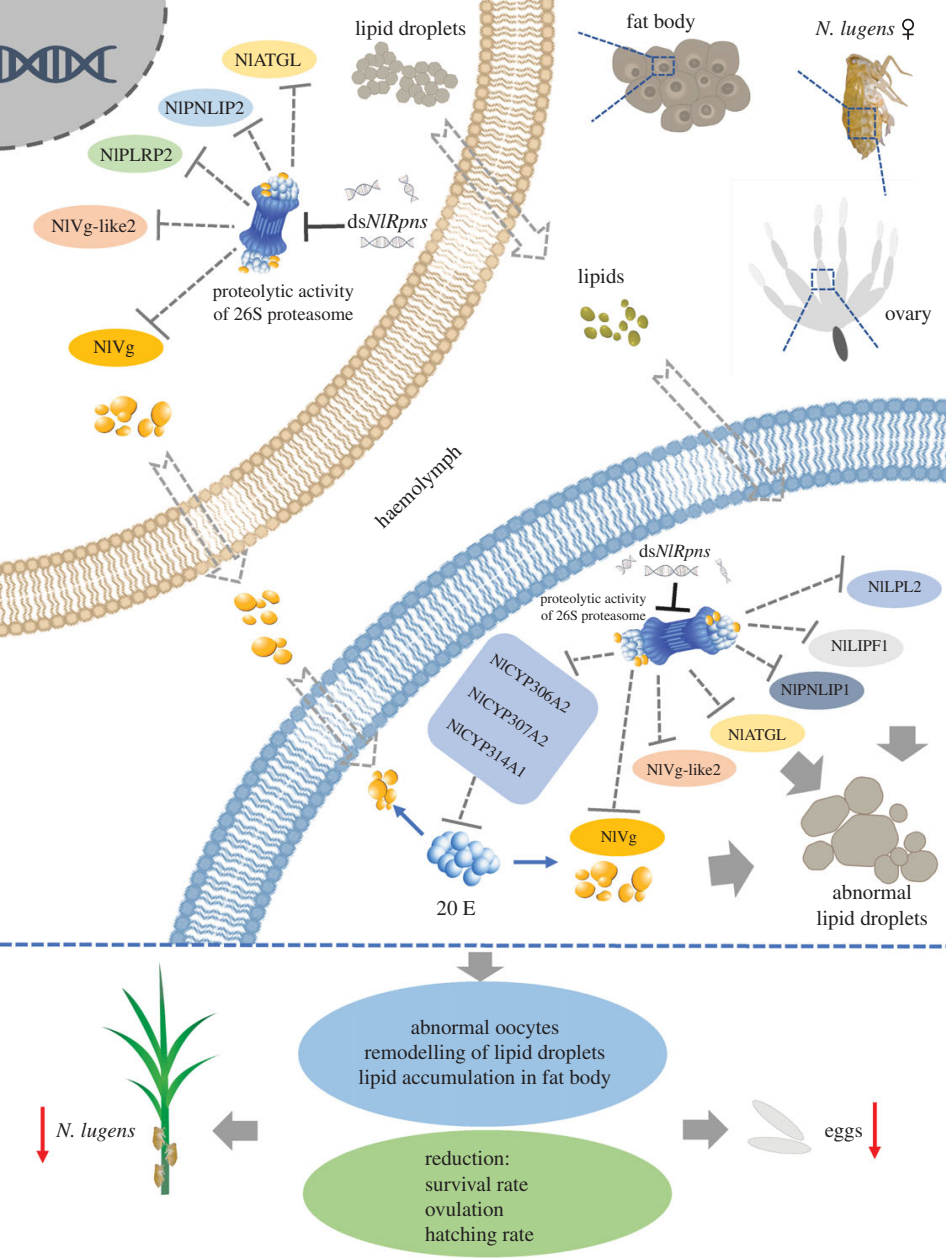
Schematic diagram of *NlRpn* affecting female reproduction in *N. lugens*. Knockdown of *NlRpn*s decreased the proteolytic activity of proteasome in *N. lugens* and impeded the transcription of *Vg*, *Vg-like* and lipid-metabolizing genes, including *adipose triglyceride lipase* (*NlATGL*), *pancreatic triacylglycerol lipase 1* (*NlPNLIP1*), *lipoprotein lipase-like 2* (*NlLPL2*), *gastric triacylglycerol lipase-like1* (*NlLIPF1*), *pancreatic lipase-related protein 2* (*NlPLRP2*), and *pancreatic triacylglycerol lipase 2* (*NlPNLIP2*) in fat bodies and/or ovaries. *NlRpn*s are also related to the expression of the 20E biosynthetic enzyme genes *NlCYP307A2*, *NlCYP306A2*, and *NlCYP314A1* in female ovaries. Knockdown of *NlRpn*s affects Vg synthesis in the fat body and utilization in the developing oocytes, disrupts lipid metabolism in the fat body and oocyte, and also inhibits 20E synthesis in the ovaries, leading to failure of oocyte maturation and development.

There is crosstalk between ecdysteroidogenesis, vitellogenesis and lipogenesis in reproduction in female insects. The regulatory pathways differ depending on the reproductive strategies adopted by various insects [60]. In *D. melanogaster*, 20E signalling regulates vitellogenesis and oogenesis in the presence of sufficient nutritional resources. In addition, 20E has been linked to lipid accumulation in oocytes during oogenesis [60,64]. In the present study, we reported that *Rpns* are involved in female reproduction in a model hemipteran insect. We proposed a regulatory mechanism for the roles of *Rpn*s in ovarian development in *N. lugens*. Furthermore, crosstalk between 20E, Vg, lipids and Rpns was hypothesized (figure 14). In summary, Vg and lipid (i.e. triglycerides) production is associated with the proteolytic activity of the proteasome in the ovaries and fat bodies. Knockdown of *NlRpn*s decreased proteasome activity, repressed *Vg* and *lipase* expression, reduced triglyceride content, decreased the Vg protein level and disrupted lipid metabolism in the ovaries, thereby leading to the defective absorption and utilization of nutrients in developing oocytes. Decrease of the proteolytic activity of the proteasome also decreased *halloween* expression and reduced 20E synthesis in the ovaries. Furthermore, insufficient levels of lipids, Vg and 20E in the ovaries disrupted oocyte maturation and development. Therefore, the proteolytic activity of the proteasome is required for insect survival, ovarian development and oocyte maturation, and our results point to the importance of the proteolytic activity of the proteasome in reproduction.

## 4. Material and methods

### 4.1. Insects

*Nilaparvata lugens* were originally collected from a rice field in the Huajiachi Campus of Zhejiang University, Hangzhou, China in 2008 and subsequently maintained in our laboratory on a diet of fresh rice seedlings (*Oryza sativa* strain Xiushui 110) at 26 ± 0.5°C and 50 ± 5% humidity under a 16 L : 8 D photoperiod, as described previously [44].

### 4.2. Bioinformatics analysis

*NlRpn* and *NlRac* sequences were searched against the *N. lugens* genome (GenBank accession number AOSB00000000 under BioProject PRJNA177647) and transcriptome (accession number SRX023419) in the Sequence Read Archive database (http://www.ncbi.nlm.nih.gov/sra) using the National Center for Biotechnology Information (NCBI) reference sequences. The amino acid sequences were predicted using DNASTAR Lasergene EditSeq (https://www.dnastar.com/software/lasergene/) and amino acid sequence alignments were carried out using ClustalX (http://www.clustal.org/). Theoretical molecular weights and isoelectric points were calculated using ExPASy (http://web.expasy.org/protparam/). The deduced domain structures were determined using SMART (http://smart.embl.de/), Pfam (http://pfam.xfam.org/) and NCBI (http://www.ncbi.nlm.nih.gov/). A phylogenic tree was constructed by the maximum-likelihood method using Mega X and phylogenetic relationships were determined by bootstrap analysis with values of 1000 trials.

### 4.3. Sample collection for developmental stage- and tissue-specific expression analyses

To investigate the expression levels of *NlRpn* and *NlRac* genes at different developmental stages, we collected samples from eggs, 1st, 2nd, 3rd, 4th and 5th instar nymphs, and adult males and females. For tissue-specific analysis, we dissected fat body, ovary, brain, cuticle, salivary gland and gut tissues from adult females under an S8AP0 stereomicroscope (Leica Microsystems GmbH, Wetzlar, Germany), and washed them quickly in diethylpyrocarbonate-treated NaCl/Pi solution (pH 7.4), as described previously [65].

### 4.4. Quantitative real-time polymerase chain reaction (qRT-PCR)

Total RNAs were extracted from each developmental stage and each tissue using an RNAiso Plus Kit (TaKaRa, Dalian, China). The concentrations of the total RNAs were determined using a NanoDrop 2000 spectrophotometer (Thermo Fisher Scientific, Bremen, Germany). The concentration of each RNA sample was adjusted with RNase-free water to 1 µg µl^−1^, and 1 µg of starting RNA was used for reverse transcription in a 20 µl reaction using a Hiscript^®^ II Q RT SuperMix for qPCR (+gDNA wiper) Kit (Vazyme, Nanjing, China) to remove any contaminating genomic DNA. A no-template control (RNA without reverse transcriptase) was used to detect any contamination. qRT-PCR was carried out on a CFX Connect™ Real-Time System (Bio-Rad, Hercules, CA, USA) using ChamQTM SYBR® Color qPCR Master Mix (Vazyme) under the following reaction programme: denaturation at 95°C for 3 min followed by 40 cycles at 95°C for 15 s and 55°C for 30 s. The gene-specific primers were designed using the Primer Premier 6.0 program based on the *N. lugens* transcriptomic sequences (electronic supplementary material, table S2). The use of reference genes as internal controls is the most appropriate normalization strategy for achieving the reliable qRT-PCR assay [66]. In our previous study, the use of *N. lugens* housekeeping *18S ribosomal RNA* (*18S rRNA*) and *β-actin* genes (GenBank accession nos. JN662398 and XP_022202043) has been validated for their stable expressions in *N. lugens* tissues and developmental stages [42,44,45,67]. In this study, the *N. lugens 18S rRNA* and *β-actin* genes were used as internal controls and the results were normalized to the expression levels of the two internal genes, respectively. The relative quantitative method (ΔΔ*C_t_* method, *C_t_* is the threshold cycle) was used to evaluate the relative differences in the transcript levels as described previously [42,44,45,67]. Namely, the following equation was used: Δ*C_t_* = the *C_t_* of specific gene—the *C_t_* of an internal gene (*18S rRNA* or *β*-*actin*). Three biological replicates were performed.

### 4.5. Double-stranded RNA synthesis and RNA interference

The open reading frame of each *NlRpn* or *NlRac* around 500 base pairs was amplified and cloned into the pMD-19 T vector (TaKaRa). dsRNA were synthesized *in vitro* using PCR-generated DNA templates using a T7 High Yield RNA Transcription Kit (Vazyme). The specific primers used to generate the DNA templates are shown in the electronic supplementary material, table S3. Newly emerged adult females were anaesthetized with carbon dioxide and microinjected with approximately 250 ng of ds*NlRpn* or ds*NlRac* using a FemtoJet microinjection system (Eppendorf-Netheler-Hinz, Hamburg, Germany). Insects injected with GFP dsRNA from *Aequorea victoria* were used as controls. The treated insects were reared on fresh rice seedlings at 26 ± 0.5°C and 50% ± 5% humidity under a 16 L : 8 D photoperiod. Phenotypes were observed following RNAi.

For examining the effects of depleting specific *NlRpn*s and *NlRac*s on other *NlRpn* and *NlRac* gene expression, RNAi was performed by injecting each ds*NlRpn* or ds*NlRac* into newly emerged adult females. Total RNAs were extracted from the whole bodies of the female adults on the 5th day after RNAi and used as starting RNAs for qRT-PCR analysis as described in Materials and methods, §4.4. dsGFP-injected insects were used as controls.

### 4.6. Determination of lipid droplet accumulation in oocytes of *Nilaparvata lugens* ovaries

Ovaries from adult females on the 5th day after emergence were used to determine lipid droplet accumulation. Intracellular lipid droplets were visualized by staining ovaries with Nile red (Sangon Biotech, Shanghai, China) [68]. Briefly, ovaries were dissected from adult females and washed three times in 1 × phosphate-buffered saline (PBS), and then fixed in 4% paraformaldehyde for 30 min at room temperature. After washing three times with PBS containing 0.1% Triton X-100 (PBST), the ovaries were incubated in 0.1% PBST buffer with 1 µg ml^−1^ Nile red solution for 90 min at room temperature to stain the lipid droplets. For nuclear staining, the ovaries were incubated for 15 min in 1 µg ml^−1^ 4′,6-diamidino-2-phenylindole (DAPI; Thermo Fisher Scientific) at room temperature followed by washing with 0.1% PBST three times. Finally, the ovaries were washed twice with 1 × PBS and transferred to microslides. Lipid droplets were observed under an LSM 800 confocal microscope (Carl Zeiss MicroImaging, Göttingen, Germany). The absorption and emission wavelengths for the lipid droplets and nuclei were 550/570 nm and 358/461 nm, respectively. The confocal images were analysed with ZEN 2.3 software (Carl Zeiss MicroImaging) and the diameters of the lipid droplets were measured using ImageJ 1.52a software (National Institutes of Health, Maryland, USA).

### 4.7. Detection of Vg in ovaries by western blotting assay

RNAi was conducted by injecting newly emerged females with each ds*NlRpn* or ds*NlRac*. Ovaries were dissected at the 5th dpi. dsGFP-injected insects were used as controls. The ovaries were washed three times with ice-cold PBS solution (137 mM NaCl, 2.68 mM KCl, 8.1 mM Na_2_HPO_4_, 1.47 mM KH_2_PO_4_, pH 7.4) and homogenized, followed by adding 6 × protein loading buffer and boiling for 15 min. The proteins were separated by sodium dodecyl sulfate-polyacrylamide gel electrophoresis at 80 V for 2 h and transferred to polyvinylidene fluoride membranes (Merck KGaA, Darmstadt, Germany) at 60 V for 2 h. Blots were probed with mouse primary antibody (1 : 5000 dilution) against *N. lugens* Vg (prepared in our laboratory) and detected using goat anti-mouse IgG-conjugated horseradish peroxidase antibody (Genscript, Nanjing, China) at a dilution of 1 : 5000. Western blot signals were visualized using a Chemiluminescence Detection Kit (Bio-Rad) and photographed with the Molecular Imager^®^ ChemiDoc™ XRS + System (Bio-Rad). The β-actin polyclonal rabbit primary antibody was used as a loading control [69].

### 4.8. Measurement of triglycerides in ovaries and fat bodies

Ovaries and fat bodies were dissected from ds*NlRpn*- or ds*NlRac*- and dsGFP-injected females at the 5th day after emergence. Following washing three times in 1× PBS, the ovaries and fat bodies were homogenized separately in lysis buffer and kept at room temperature for 10 min, and the homogenates were then heated for 10 min at 70°C and centrifuged for 5 min at 2000*g*. The triglyceride contents of the supernatants were measured using a Tissue Triglyceride Assay Kit (Applygen Technologies, Beijing, China) and protein concentrations were analysed using a Bicinchoninic Acid (BCA) Protein Assay Kit (Thermo Fisher Scientific), according to the manufacturer's instructions.

### 4.9. Functional analysis of *NlRpn* and *NlRac* genes during ovulation and egg hatching

Ovulation and egg hatching experiments were conducted according to Lou *et al.* [16]. Briefly, newly emerged females were microinjected with each ds*NlRpn* or ds*NlRac* and dsGFP, respectively. Brachypterous females have higher fecundity than macropterous females, and we therefore used brachypterous female *N. lugens* for ovulation experiments in this study. Newly emerged brachypterous females were injected with the target dsRNAs and reared on fresh rice seedlings for 1 day. To ensure successful mating, a single ds*NlRpn*- or ds*NlRac*-injected or dsGFP-injected control adult female was mated with two non-injected adult males in a long glass tube containing three-leaf stage fresh rice seedlings (9 ± 0.5 cm long) at 26 ± 0.5°C and 50% ± 5% relative humidity under a 16 L : 8 D photoperiod for 6 days. The adult insects were then removed and hatched nymphs were observed and counted at 24 h intervals, and were removed from the rice seedlings 11 days later. Unhatched eggs were dissected and counted. Biological replicates were carried out for each mating (*n* = 17–20 females × males).

### 4.10. Measurement of 20E in *Nilaparvata lugens* adult females

We previously determined the 20E contents of *N. lugens* nymphs and adults [45]. In this study, 20E contents were measured in adult females after *NlRpn* and *NlRac* RNAi. Briefly, newly emerged adult females were injected with each ds*NlRpn* or ds*NlRac*, with dsGFP-injected insects as controls. The 20E content was measured in the whole body at 120 h after emergence, according to Nakaoka *et al*. [70] with some modifications, as described in Zhou *et al.* [45].

### 4.11. *In vitro* proteasome activity assay

Each newly emerged adult female was injected with 250 ng of ds*NlPSMB5* or 250 ng of a mixture of ds*NlRpn*s and ds*NlRac*s at equal quality ratio (each dsRNA 100 µg in a mixture of dsRNAs). The adult females were collected at the 5th day after RNAi. dsGFP-injected insects were used as controls. The *in vitro* proteasome activity assay using the fluorogenic peptide *N*-Succinyl-Leu-Leu-Val-Tyr-7-amido-4-methylcoumarin (Suc-LLVY-AMC; Sigma, Missouri, USA) was performed with some modifications to measure the chymotrypsin-like proteasome activity, which has been reported to be the most specific substrate to measure proteasome activity as described previously [71,72]. Briefly, 20 mg of the whole bodies of adult females were homogenized in 200 µl TSDG buffer (10 mmol l^−1^ Tris/HCl, 1.1 mmol l^−1^ MgCl_2_, 10 mmol l^−1^ NaCl, 0.1 mmol l^−1^ EDTA, 1 mmol l^−1^ DTT, 2 mmol l^−1^ ATP, 10% (v/v) glycerol, pH 7.0). The homogenates were centrifuged at 4°C at 20 000*g* for 10 min and the supernatants were used for an *in vitro* proteasome activity assay. The fluorogenic peptide substrate Suc-LLVY-AMC was dissolved in 100% DMSO as a stock solution of 10 mmol l^−1^. Ten microlitres of the supernatants were added to 20 µl of PBS and 30 µl of 200 µmol l^−1^ Suc-LLVY-AMC solution that was diluted 50 times with TEAD buffer (20 mmol l^−1^ Tris/HCl, 1 mmol l^−1^ EDTA, 1 mmol l^−1^ DTT, pH 7.2) from the Suc-LLVY-AMC stock solution and incubated for 1 h at 25°C. The fluorescence intensities were measured at 10 min intervals for 60 min under a multifunctional microplate reader Varioskan LUX (Thermo Fisher Scientific). The absorption and emission wavelengths for the Suc-LLVY-AMC were 380/440 nm. 10 µl TSDG buffer was added to 20 µl of PBS and 30 µl of 200 µmol l^−1^ Suc-LLVY-AMC solution and used as a negative control.

### 4.12. Statistical analysis

Ovulation experiment results were calculated from at least 17 biological replicates; the other assays were from at least three biological replicates. The significance values were determined using Student's *t*-test (*p < 0.05; **p < 0.01) with GraphPad Prism 8 software (San Diego, CA, USA).

## Data accessibility

This article has no additional data.
